# A hybrid approach reveals the allosteric regulation of GTP cyclohydrolase I

**DOI:** 10.1073/pnas.2013473117

**Published:** 2020-11-23

**Authors:** Rebecca Ebenhoch, Simone Prinz, Susann Kaltwasser, Deryck J. Mills, Robert Meinecke, Martin Rübbelke, Dirk Reinert, Margit Bauer, Lisa Weixler, Markus Zeeb, Janet Vonck, Herbert Nar

**Affiliations:** ^a^Medicinal Chemistry, Boehringer Ingelheim Pharma GmbH & Co. KG, 88397 Biberach an der Riss, Germany;; ^b^Structural Biology, Max Planck Institute of Biophysics, 60438 Frankfurt am Main, Germany

**Keywords:** allosteric regulation, GTP, cryo-EM, X-ray crystallography, STD-NMR

## Abstract

Guanosine triphosphate (GTP) cyclohydrolase I (GCH1) catalyzes the conversion of GTP to dihydroneopterin triphosphate (H2NTP), the initiating step in the biosynthesis of tetrahydrobiopterin (BH4). Besides other roles, BH4 functions as cofactor in neurotransmitter biosynthesis. The BH4 biosynthetic pathway and GCH1 have been identified as promising targets to treat pain disorders in patients. The function of mammalian GCH1s is regulated by a metabolic sensing mechanism involving a regulator protein, GCH1 feedback regulatory protein (GFRP). GFRP binds to GCH1 to form inhibited or activated complexes dependent on availability of cofactor ligands, BH4 and phenylalanine, respectively. We determined high-resolution structures of human GCH1−GFRP complexes by cryoelectron microscopy (cryo-EM). Cryo-EM revealed structural flexibility of specific and relevant surface lining loops, which previously was not detected by X-ray crystallography due to crystal packing effects. Further, we studied allosteric regulation of isolated GCH1 by X-ray crystallography. Using the combined structural information, we are able to obtain a comprehensive picture of the mechanism of allosteric regulation. Local rearrangements in the allosteric pocket upon BH4 binding result in drastic changes in the quaternary structure of the enzyme, leading to a more compact, tense form of the inhibited protein, and translocate to the active site, leading to an open, more flexible structure of its surroundings. Inhibition of the enzymatic activity is not a result of hindrance of substrate binding, but rather a consequence of accelerated substrate binding kinetics as shown by saturation transfer difference NMR (STD-NMR) and site-directed mutagenesis. We propose a dissociation rate controlled mechanism of allosteric, noncompetitive inhibition.

## Significance

We present a comprehensive structural study, which shows the human GCH1 and GCH1−GFRP complexes in all states (apo, ligand bound, partially and fully inhibited). We observed local rearrangements in the allosteric pocket upon BH4 binding, which result in drastic changes in the quaternary structure of the enzyme leading to a more compact, tense form in the inhibited protein. Inhibition of the enzymatic activity is not a result of hindrance of substrate binding, but rather a consequence of accelerated substrate binding kinetics as shown by STD-NMR and site-directed mutagenesis. We propose a dissociation rate-controlled mechanism of allosteric, noncompetitive inhibition, which could stimulate further research toward the development of GCH1 inhibitors to treat neuropathic and inflammatory pain disorders.

Guanosine triphosphate (GTP) cyclohydrolase I (GCH1) (EC:3.5.4.16) catalyzes the conversion of GTP to dihydroneopterin triphosphate (H2NTP). This reaction is the first and rate-limiting step involved in the de novo synthesis of tetrahydrobiopterin (BH4) (1). BH4 plays key roles in phenylalanine catabolism and the biosynthesis of serotonin and catecholamine-type neurotransmitters like dopamine or norepinephrine by functioning as an essential cofactor for hydroxylases of the aromatic amino acids phenylalanine, tyrosine, and tryptophan (2, 3). Further, BH4 is cofactor for the family of nitric oxide synthases (4), which produce the cellular signaling molecule nitric oxide that helps to modulate vascular tone and insulin secretion and affects inflammation as well as the regulation of immune responses (5).

Several lines of evidence, including human genetic data that show that a GCH1-deficient haplotype is pain resistant, suggest that selective inhibition of GCH1 is an attractive target to treat neuropathic and inflammatory pain disorders (6–8). Abnormalities in the control mechanisms of GCH1 or the activities in other enzymes of its biosynthetic pathway leads to BH4 deficiency, which is linked to a variety of vascular diseases such as diabetes, atherosclerosis, and hypertension (9–14) and to neurological disorders, including Parkinson’s disease (15, 16). These examples impressively show the serious consequences of nonphysiological levels of BH4. Nature therefore evolved a highly regulated mechanism of BH4 homeostasis.

In a seminal paper (17) by Harada et al., the molecular basis of BH4 homeostasis was uncovered and shown to involve GCH1 and a regulatory protein, now known as GTP-cyclohydrolase-I-feedback-regulatory protein (GFRP), which simultaneously functions as a positive and negative regulator of GCH1 (17). The effects of GFRP on GCH1 occur via formation of heteromeric protein complexes between GCH1 and GFRP, which are dependent on the intracellular concentrations of the effector molecules phenylalanine or BH4. Elevated phenylalanine levels lead to stimulation of GCH1 activity, whereas BH4, the end product of the biosynthesis pathway, inhibits GCH1 in a feedback inhibition type mode (18). Mammalian GCH1 shows cooperative enzymatic activity. Complex formation with GFRP-Phe leads to increased activity at lower substrate concentrations and eliminates substrate cooperativity. Conversely, GCH1 alone is allosterically inhibited by BH4. In the presence of GFRP, the inhibitory effect of BH4 is boosted and occurs at lower, physiologically relevant BH4 concentrations. The GCH1−GFRP system can therefore be regarded as a metabolic sensor that establishes BH4 and aromatic amino acid homeostasis.

The human GCH1 sequence comprises 250 amino acids and forms a 270-kDa, D5-symmetric homodecameric functional enzyme complex in solution (19, 20). GFRP occurs as a pentamer of 50 kDa (5 × 10 kDa). GCH1−GFRP complexes consist of one GCH1 decamer flanked by two pentameric GFRP complexes. Association is along the particle fivefold axes, and the complexes are ∼370 kDa in size (21).

Structural information on GCH1 was first obtained on the *Escherichia coli* enzyme (19, 22, 23); later structures of the human GCH1 (24) and rat GCH1−GFRP complexes (18, 25) were determined. The structures revealed the subunit fold and quaternary structure of the functional complex and established GCH1 as a Zn(II)-dependent hydrolase. The X-ray structures of stimulatory and inhibitory rat GCH1−GFRP complexes show that phenylalanine binds to a surface pocket on GFRP close to the protein−protein interaction interface with GCH1, whereas BH4 binds to an allosteric pocket on GCH1 close to the GFRP interface (18, 25, 26). Structural differences between stimulatory and inhibitory complexes were found to be minor. The medium resolution (2.8 Å) of the studies and the circumstance that, for this particular case, folding and unfolding events play a major role, and are impacted by crystal packing artifacts, did not allow for detailed insights into the structural basis of allosteric control mechanisms.

We therefore decided to investigate this unresolved issue. First, we determined the structures of human GCH1−GFRP complexes by cryoelectron microscopy (cryo-EM) at high resolution. Further, we conducted elaborate structural, enzyme kinetics, biophysics, and mutagenesis studies on human GCH1 and used the results in conjunction with the cryo-EM data to identify the key structural features responsible for the allosteric inhibition of GCH1.

## Results

We suspected, from analysis of the rGCH1−rGFRP crystal structures, that important functional regions on the protein surface undergo order−disorder transitions on the trajectory from active to inactive or substrate-bound states and could be impacted by crystal packing. Therefore, we decided to use cryo-EM as a complementary approach to resolve the structures of hGCH1−hGFRP complexes in an aqueous environment, unbiased by crystal contacts.

Further, we crystallized hGCH1 in the presence and absence of allosteric and active site ligands. In order to allow the protein to arrange in its preferred conformation, all crystals were generated by cocrystallization. We obtained crystal structures of different conformational states and varying levels of occupancy of ligand binding. These structural snapshots helped to delineate the consequences of ligand binding to the GCH1 allosteric site and their influence on the protein conformation.

A variety of ligands were used to generate the structural information. BH4 and one 2,4-diamino-6-hydroxypyrimidine (DAHP) analog were selected as GCH1 allosteric site binders. In the literature, these are described as noncompetitive inhibitors (16). AXSP0056BS (cpd-1) was identified as an allosteric GCH1 inhibitor in an enzymatic assay with half maximal inhibitory concentration (IC50) values of 4 µM. The 8-oxo-GTP, a tightly binding, substrate analog GCH1 inhibitor, and 7-deaza-GTP, a nonhydrolyzable, more weakly inhibiting analog were used as binders to the active site of GCH1.

Table 1 shows the ensemble of generated structures, the structure determination method, and the names with which we will refer to them in the following. The data collection and refinement statistics of all crystals are summarized in *SI Appendix*, Table S1, while the EM data collection, processing, and structure validation parameters are stated in *SI Appendix*, Table S2.

**Table 1.** Summary of obtained crystal and EM structure



The colors indicated correspond to the colors in figures. Crystallographic data statistics are summarized in *SI Appendix*, Fig. S1. EM data collection and processing statistics are summarized in *SI Appendix*, Fig. S2.

**Fig. 1.**
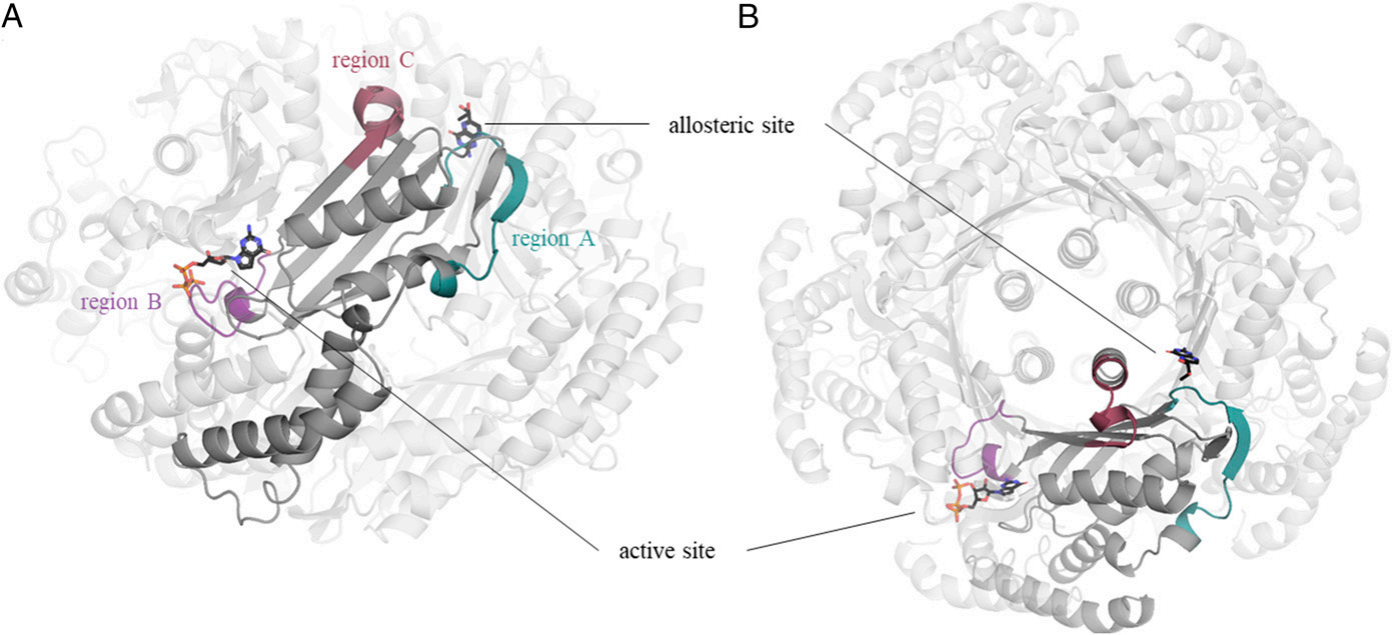
Relevant structural features on GCH1. The homodecamer of GCH1 is colored in light gray. One GCH1 monomer is highlighted in darker gray. For further analysis, relevant regions on GCH1 are colored according to the description in Table 2. The locations of the active and the allosteric sites are indicated by the ligands GTP and BH4, respectively, in stick representation (black). (*A*) Side view of GCH1. (*B*) Top view of GCH1 depicting the location of the five-helix bundle and β-barrel in the center of the protein, which are formed form by α-helices or β-strands from five protomers.

In the following analysis of the protein structures, we will focus on the regions A through C, which are shown in Fig. 1 and Table 2. These regions show the most pronounced conformational changes. As expected for allosteric enzymes such as GCH1, these regions are predominantly located at the periphery of GCH1 monomers, forming the interface between the individual protomers.

**Table 2.** Relevant structural features on GCH1

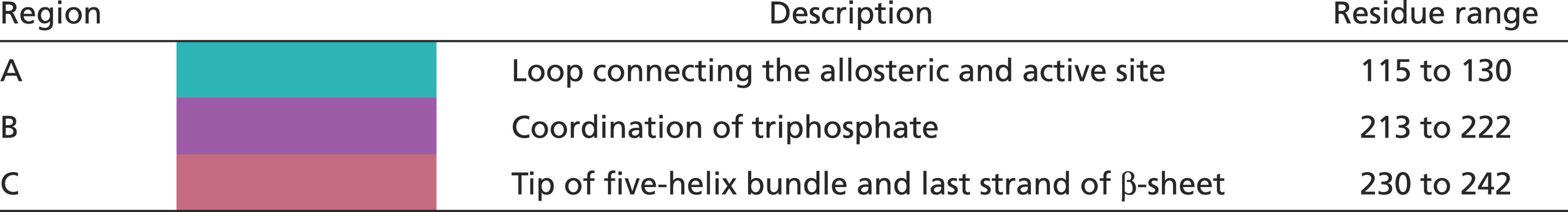

The colors indicated correspond to the colors in Fig. 1. Residue numbers correspond to the human GCH1 sequence.

### Cryo-EM Structures of Human GCH1−GFRP Complexes Reveal Dramatic Quaternary Conformational Changes and Order−Disorder Transitions.

Due to their size and symmetry, hGCH1−hGFRP complexes are well suited for high-resolution studies using cryo-EM. Structures of the inhibitory (EM_hGCH1-hGFRP+BH4) and the stimulatory (EM_hGCH1-hGFRP+Phe+active) hGCH1−hGFRP complexes were generated using single-particle cryo-EM. Suitable protein material for the cryo-EM studies was generated by complex formation in the presence of effector molecules, after separate expression and purification of both protein components, GFRP and GCH1. For the inhibitory complex, an hGCH1−hGFRP−BH4 ternary complex was produced by mixing the components (GCH1, GFRP, BH4) and purification via size exclusion chromatography (SEC). The stimulatory hGCH1−GRFP complex was formed in the presence of phenylalanine and 8-oxo-GTP, a tightly binding, substrate analog GCH1 inhibitor. The identification of suitable buffer conditions for both complexes was key to the preparation of high-quality grids.

**Fig. 2.**
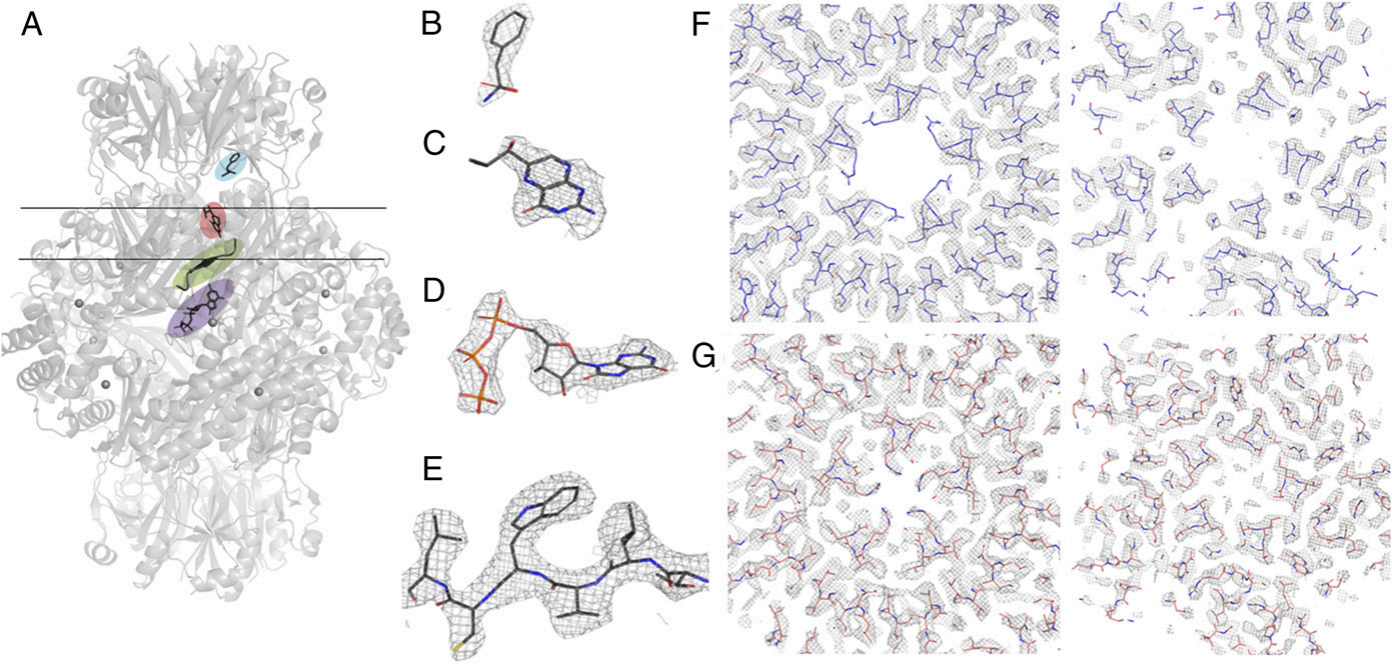
Overview of the architecture of the GCH1−GFRP complex and details of the EM maps of the stimulatory and inhibitory complexes. (*A*) The hGCH−hGFRP complex (gray) depicting the binding site for the substrate (purple) and the allosteric effector molecules phenylalanine (blue) and BH4 (salmon), as well as the position of region A (green), which is the loop connecting the active and allosteric sites. Observed EM density (black mesh) of the ligands (*B*) phenylalanine, (*C*) BH4, and (*D*) 8-oxo-GTP. (*E*) Quality of cryo-EM density map. Selected region showing the fit of the derived atomic model to the cryo-EM density map (black mesh). For *F* and *G*, both EM structures were aligned before images of the cross-sections along the *y* axis were taken. The lines in *A* correspond to the height of the slices. The upper line in *A* marks the cutting height of the cross-section in *F* and *G*, *Left*, while the lower line in *A* represents the height of the cross-section in *F* and *G*, *Right*. *F* shows a section through GCH1 of the stimulatory complex, while *G* shows the equivalent section for the inhibitory complex.

For both complexes, ∼3,000 micrographs were recorded and processed in Relion-3.0. The inhibitory and stimulatory protein complexes were reconstructed applying D5 symmetry, yielding maps with resolution of 2.9 and 3.0 Å, respectively, which allowed building of atomic models (Fig. 2*E* and *SI Appendix*, Fig. S1).

**Fig. 3.**
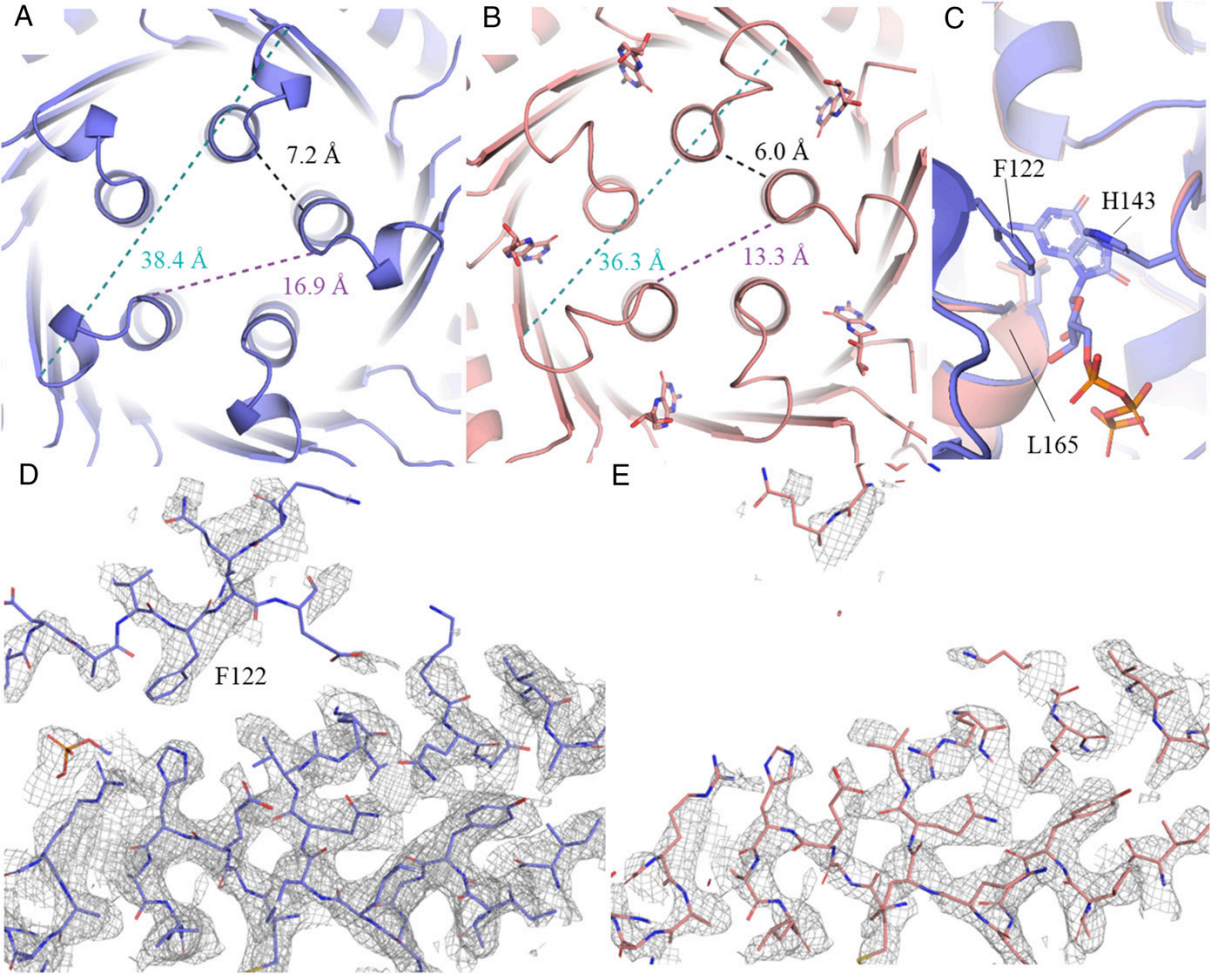
Details of the stimulatory (blue) and inhibitory (salmon) EM structures reveal dramatic quaternary conformational changes and order−disorder transitions. Distance measurements show the changes of the radius of the β-barrel and the five-helix bundle for the (*A*) stimulatory (blue) and the (*B*) inhibitory (salmon) complexes. The distances were obtained by measuring the Cα backbone distances of the residues Gly232A to Gly232C, Pro238C to Pro238E, and Lys239E to Pro238A. (*C*) π-stacking of F122 against His143 of the opposite site of the active site cleft fixes region A in closed conformation in the stimulatory complex (blue). No shifting of L165 backbone and respective helix structure is observed between the inhibitory (salmon) and stimulatory (blue) complex. EM density of the (*D*) stimulatory and (*E*) inhibitory complexes shows no density for loop F122, while it is defined in the stimulatory complex, indicating a higher degree of flexibility in region A in the inhibitory complex.

The quality and resolution of the EM maps allowed for unambiguous identification of bound ligands (Fig. 2 *B*–*D*). Comparison of the cryo-EM−derived inhibitory and stimulatory GCH1−GFRP complexes reveals a significant increase in the density of the EM map at the core of the GCH1 decamer involving the central five-helix bundle and the 20-stranded β-barrel (region C) as a result of allosteric ligands and GFRP binding (Fig. 2 *F* and *G*). Looking at region C, this observation fits well with existing models of allosteric enzymes, which control their regulation via an active, mobile state (relaxed state) and an inactive, rigid state (tensed state). Helices approach each other by moving more than 1 Å, and the β-barrel diameter is reduced by 2 Å. Fig. 3 *A* and *B* shows top views of both structures and the respective distance measurements. Exactly the same conformational rearrangement is present in the rat complex structures (18), but this finding had been overlooked in the analysis of rat complexes and was not discussed by the authors of these structures.

Secondly, the EM structures reveal a drastic difference between the inhibitory and stimulatory hGCH1−hGFRP complexes in the structure and flexibility of the surface-lining loop 115 to 130 (region A). This loop is the only direct connection between the active and the allosteric site, and it is in close proximity to both sites. F122 and its close periphery appears well ordered and in a distinct closed conformation in the active site ligand-bound state of the stimulatory complex, while it is not visible in the densities after reconstruction of the inhibitory complex (Fig. 3 *C* and *D*). For a valid comparison of both EM maps, their contour levels were adjusted at regions which are similarly well defined in both structures (Cα 236 to 249, contour level = 1.7 to 1.9 rmsd). In the inhibitory complex, a high degree of flexibility is responsible for the apparent disorder. The loop does not seem to be held in place by any relevant interaction with the surrounding protein structure. In the stimulatory complex, however, the loop is ordered and visible in the maps. F122 seems to act as a clasp, holding the loop in position by π-stacking against His143, localized at the opposite side of the active site cleft (Fig. 3*C*).

The finding of a disorder−order transition in GCH1 is in contrast to the current model discussed in the literature. Based on findings in the rGCH1 complex X-ray structures, the authors discuss a major conformational change involving F122 (F113 in the rat GCH1 sequence) and L165 (L156 in rGCH1) (18). F122 is in an outward orientation in the inhibitory complex, leading to a solvent-exposed active site, whereas it is inward oriented in the stimulatory complex, leading to a closed, solvent-shielded active site. In addition, they discuss a displacement of L165 in the inhibitory complex that decreases the depth of the GTP pocket by shifting toward the active site, thereby permitting substrate binding. Close inspection of their experimental data shows that region A is involved in direct crystal contacts in some protomers of both rat structures. It is obvious that direct crystal packing can influence conformations of surface lining regions. However, crystal packing artifacts not only have an influence on the conformation of the affected protomer region but can also change the energy landscape and conformations of neighboring subunits, especially in highly symmetric, cooperative and multimeric proteins with large subunit interfaces. As the EM data were recorded in a solute environment, we presume that, in the crystal structure, region A was artificially locked in a more rigid state and that the loop is in fact disordered in solution. Additionally, in our superpositions of the human protein structures (Fig. 3*C*), L165 is not displaced, and the active site machinery is, in fact, fully structurally conserved in both complexes and should allow for substrate binding.

### Crystal Structures of Active, Substrate-Bound, and Allosterically Inhibited GCH1 Reveal Atomic Details of the Transition from Active to Inhibited Conformations.

In parallel to the cryo-EM studies, we wanted to generate additional information on as of yet unknown conformational states of GCH1 alone, in the absence of GFRP. We chose to utilize a set of active site and allosteric site inhibitors to lock GCH1 in active and inhibited conformations.

**Fig. 4.**
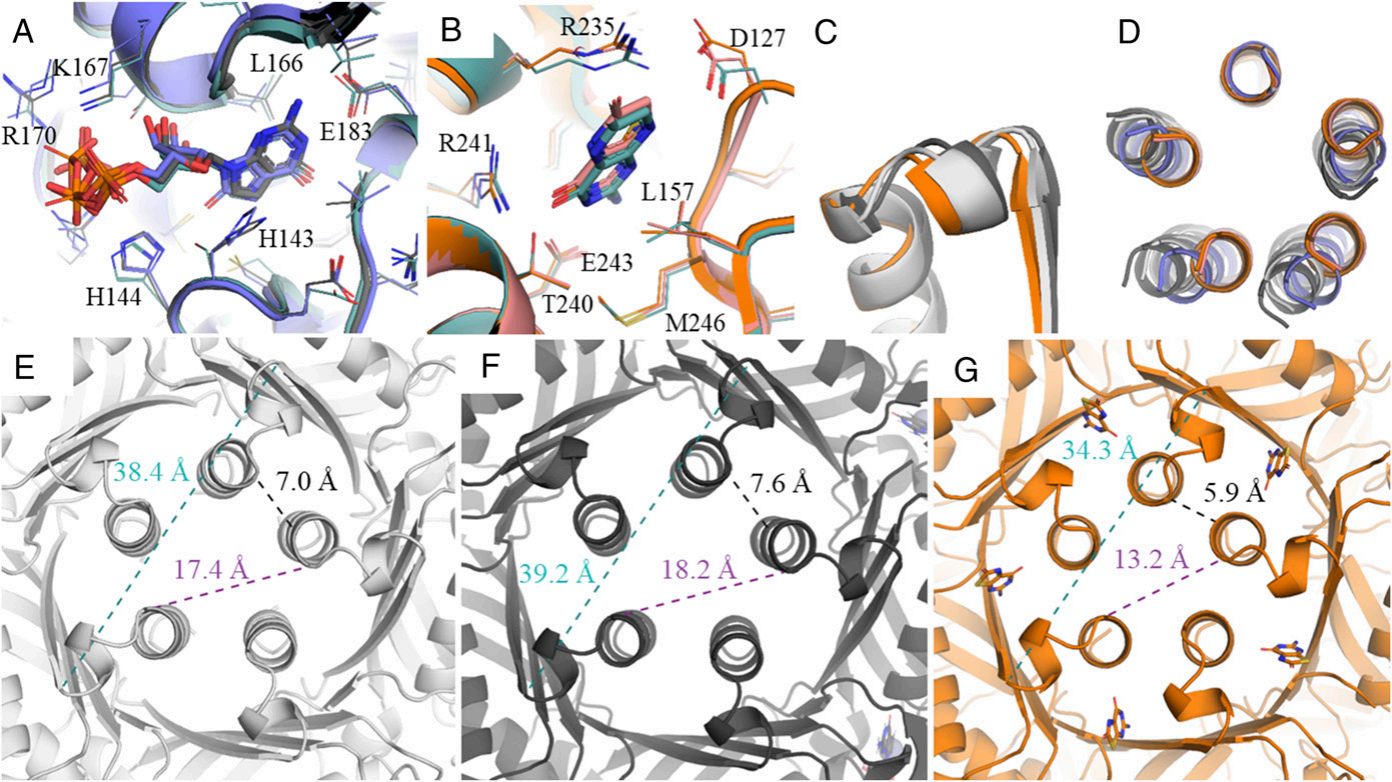
Overall conformational changes upon binding of orthosteric and allosteric ligands. (*A*) Comparison of substrate analog binding mode in EM_hGCH1+hGFRP+Phe+active (blue), the xtal_GCH1+ active (dark gray), and the published substrate-bound *E. coli* structure (1A8R) (turquoise). (*B*) Inhibitor recognition in the allosteric pocket on GCH1 of the EM_hGCH1+hGFRP+allosteric (salmon) structure, the xtal_GCH1+allosteric (orange) structure, and the published structure of the inhibitory rat complex (1WPL) (turquoise). (*C*) Movements of region A toward the interior of the protein upon binding of allosteric inhibitors and opposing motion upon binding of substrate analogs. Comparison between the xtal_GCH1 (light gray), xtal_GCH1+active (dark gray), and xtal_GCH1+allosteric (orange) structures. (*D*) Five-helix bundle overlay of all human GCH1 and GCH1−GFRP structures, showing a perfect overlay of the EM_hGCH1+hGFRP+allosteric (salmon) and the xtal_GCH1+allosteric (orange) structures, as well as the xtal_GCH1 (light gray) and xtal_GCH1+active (dark gray) structures. The structures cluster into three distinct conformations: active (light gray, dark gray), stimulated (blue), and inhibited (salmon, orange). The coloring of the structures is according to the colors listed in Table 1. Distance measurements show the changes of the radius of the β-barrel and the five-helix bundle between (*E*) xtal_GCH1 (light gray), (*F*) xtal_GCH1+active (dark gray), and (*G*) xtal_GCH1+allosteric (orange) structures.

We determined structures of the human GCH1 in the absence (xtal_GCH1) and presence (xtal_GCH1+active) of the substrate analog 7-deaza-GTP and in the presence of the allosteric inhibitor cpd-1 (xtal_GCH1+allosteric and xtal_GCH1+allosteric2). Xtal_GCH1 is a redetermination of the previously determined apo-hGCH1 structure (24). Fig. 4*A* shows the overlay of bound substrate analogs, and Fig. 4*B* shows the binding mode of inhibitors binding to the allosteric site. Small-molecules recognition does not differ from the observed binding mode in published *Escherichia coli.* (1a8r; eGCH1[H112S]+GTP) and rat (1wpl; rGCH1-rGFRP+BH4) crystal structures (18, 27).

Using suitable superpositions of GCH1 monomers and pentameric and decameric ensembles, we analyzed local and global structural changes along the path from unliganded, basally active enzyme to a substrate analog bound, catalytically competent transition state form as well as to partially and fully allosterically inhibited structures. Further, we compare these structures with the cryo-EM structures to arrive at a holistic view on a series of conformational states accessible to mammalian GCH1.

### Protein Flexibility.

The observation of region A flexibility described for the cryo-EM data is fully supported by the crystallographic data (RMSD = 0.4 Å to 0.7 Å GCH1 [Cα monomer] EM vs. X-ray structures). The same degree of disorder seen for the inhibitory cryo-EM structure was observed for the xtal_GCH1_allosteric structure, while region A is ordered and F122 is in a defined closed state in xtal_GCH1+active. For the unliganded form xtal_GCH1, weak electron density is visible for region A that allows for tracing the main chain, suggesting that a partially flexible region A is a signature of the basally active state of GCH1. This degree of flexibility in region A, which could possibly serve as a lid for the active site, could be essential to allow substrate association.

Region B consisting of residues 213 to 222 is only weakly defined in the electron densities in all structures without an active site ligand bound (xtal_GCH1, EM_GCH1+hGFRP+allosteric, and xtal_GCH1+allosteric). It is well defined in the other structures (xtal_GCH1+active and EM_GCH1+hGFRP+Phe+active). R216 is part of this region and, in substrate analog complexed states, coordinates the β- and γ-phosphate of GTP variants and contributes to the charge compensation of the triphosphate moiety. Locking the R216 side chain in the presence of triphosphate ligands thus leads to a stabilization of a preferred conformation of this loop.

### Effect of Substrate Binding—Stimulatory Trajectory.

Upon formation of the stimulatory complex, which implies association of phenylalanine-bound GFRP to GCH1, only minor conformational rearrangements occur. Attachment of GFRP leads to a slight twisting of the last β-strand of GCH1′s β-barrel and the central α-helix in anticlockwise direction.

The most obvious conformational change upon substrate binding occurs in region A. The partial disorder of the residue range 115 to 130 is resolved into a well-defined loop structure closing the upper ceiling of the active site pocket. This disorder−order transition is clearly seen in both the cryo-EM maps and the X-ray structures. Further, region B involving the triphosphate binding pharmacophore becomes ordered as discussed above. Finally, upon binding of 7-deaza-GTP, changes in region C, including positioning of the five-helix bundle and curvature of the β-barrel, are observed. Here, region C bends outward, toward the active site, and the protein inside expands in comparison to the apo structure (Fig. 4 *E* and *F*). This movement is opposite to the observed conformational change in the inhibitory complex.

### Effect of Allosteric Inhibitor Binding—Inhibitory Trajectory.

From one cocrystallization campaign, surprisingly, we obtained two independent structures of allosterically inhibited GCH1: one fully inhibited form (xtal_GCH1_allosteric) with all allosteric sites occupied by inhibitor, and a partially inhibited form (xtal_GCH1_allosteric2), in which only two of five allosteric pockets in each GCH1 pentamer were occupied with cpd-1.

We do not see any major differences between the cryo-EM structure of the BH4−GFRP-inhibited GCH1 and the X-ray structure of cpd-1−inhibited GCH1. In particular, the quaternary structural changes in region C are replicated in the X-ray structure, namely, the shift of the central α-helices. The shrinking of the β-barrel radius is even more pronounced, and the radius of the β-barrel decreases by 5 Å, when compared to apo GCH1 (Fig. 4 *E* and *G*). Thus, the major trigger for the large change in quaternary structure and compaction of the GCH1 interior is binding of ligands into the inhibitory allosteric pocket, independent from association of GFRP.

**Fig. 5.**
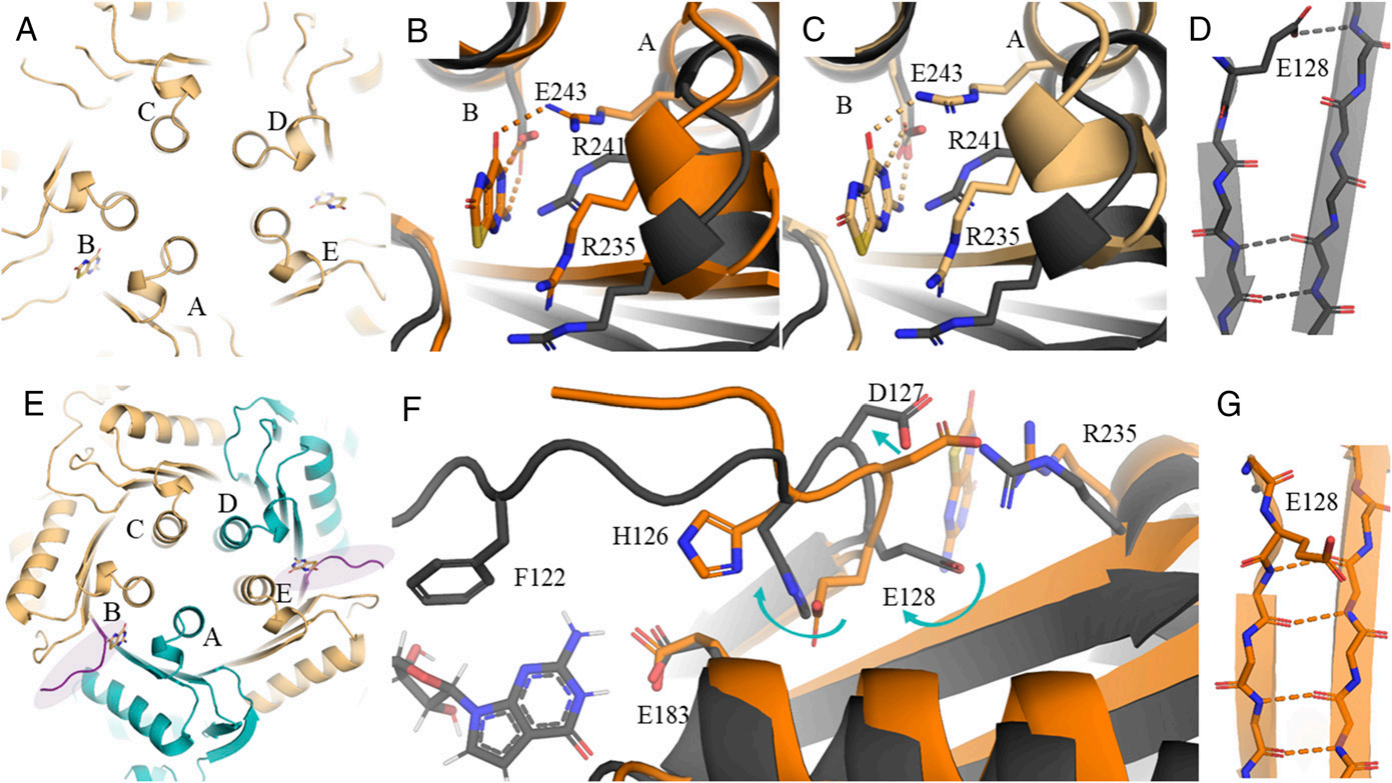
Details of conformational rearrangement upon binding of AXSP0056BS (cpd-1) to the allosteric site of GCH1. (*A*) Cartoon representation of the view of xtal_GCH1+allosteric2 shows the asymmetric rearrangement of the five-helix bundles. *B* and *C* show a top view on the allosteric site of (*B*) xtal_GCH1+allosteric2 (light orange) (chains A + B) and (*C*) xtal_GCH1+allosteric (orange) in comparison to the active structure xtal_GCH1+active (dark gray) (alignment on chain B). (*E*) Cartoon representation of the top view of xtal_GCH1+allosteric2. The regions that are in active conformations are colored in light orange, while the teal protomers show the quaternary characteristics of the inhibited conformation. The purple colored loops (protomer B and E) indicate the loop regions, in which we observed a high degree of disorder and which are therefore thought to be flexible. (*F*) Rearrangements in loop 115–130 upon binding of an allosteric inhibitor in xtal_GCH1+allosteric. (*D* and *G*) Interface between two protomers forming the β-barrel.

The partially occupied structure xtal_GCH1+allosteric2 allows delineation of the atomic details of the structural transition. Strikingly, the GCH1 structure in the xtal_GCH1+allosteric2 crystal does not adopt the fivefold symmetry as usual, but is shifted to an asymmetric state (Fig. 5*A*). Cpd-1 binds to two allosteric sites of each GCH1 pentamer, which are located in the interface of chains A and B and of chains D and E. The three remaining allosteric sites per pentamer remain unoccupied. Binding of inhibitors into the allosteric site, which is flanked by two distinct protomers, trigger different process changes in the respective subunits. Interactions of residues of one subunit (chains A or D, right) trigger conformational rearrangements of the respective subunit, while the conformation of the other subunit (chains B and E, left) does not change (Fig. 5 *A* and *E*).

Fig. 5*A* shows the top view on the allosteric site. Binding of cpd-1 induces concomitant approximation of the C-terminal helices and β-strands of subunits A and B (D and E, respectively), while distances between helices B and D, C and D, and A and E remain unchanged relative to the active state of the enzyme.

Analysis of the conformational states of all protomers show that chains A and D exhibit an inhibitory xtal_GCH1+allosteric-like state, while protomers B, C, and E are in an apo-like conformation in terms of helix bundle and β-barrel positioning and curvature (Fig. 5*E*). Region A is ordered in protomers A, C, and D, but is highly flexible in chains B and E. This observation agrees with the also drastically increased flexibility of region A in the remaining allosteric ligand-bound EM and X-ray structures.

Binding of an allosteric inhibitor to the allosteric pocket triggers a cascade of conformational changes of proximal residues. E243 (chain B) is the recognition motif for the amino pyrimidone substructure of inhibitors (Figs. 4*B* and 5 *B* and *C*). It does not need to move much in order to coordinate the 2-aminopyrimidine moiety of BH4 and analogs. In contrast, R241 (chain A) moves back and counterclockwise by 1.5 Å (alignment of xtal_GCH1+allosteric2 and xtal_GCH1+active on chains A + B) to form a hydrogen bond to the carbonyl group of BH4 or cpd-1. At the same time, the neighboring R235 (chain A) moves toward the protein interior, facilitating cation−π-stacking of its guanidine group and the biopterin heterocycle (Fig. 5*B*). Here the backbone shifts by 2 Å. Due to these rearrangements and changes in the hydrogen network, the entire backbone of residues 230 to 240 of region A shifts by 0.8 Å to 2.4 Å. From our analysis, it remains unknown whether R241 or R235 is the main trigger for conformational rearrangement or whether a joint movement of both residues is necessary.

Change of position of R235 as well as the steric requirements of the ligand BH4 in the pocket leads to further local structural changes. E128 is pushed out of its position in the active state in which it forms a hydrogen bond to the backbone NH of M230 of the C-terminal β-strand of the neighboring subunit. Removed from its old position, it allows closure of the top part of the β-barrel, which triggers the compaction of the barrel by formation of a tight hydrogen network between both neighboring strands as discussed above (Fig. 5 *D* and *G*). D127 is recruited by R235 to form a charge-reinforced hydrogen bond of their side chains. This movement completes the closure and formation of the final shape of the allosteric pocket.

These two rearrangements involving D127 and E128 trigger further changes in the structure of residues 120 to 126 that lie on the path toward the active site. In particular, H126 is displaced to a new position pointing toward E183, the guanine recognition residue in the active site (Fig. 5*F*). The exchange of positions of the H126 and E128 side chains in the inhibitory state leads to a loosening of the 115–125 loop structure by two amino acids. This may be the structural basis for increased loop flexibility observed in the inhibited protomers in the xtal_GCH1+allosteric (chains A, B, C, D, and E) and xtal_GCH1+allosteric2 structures (chains B and E).

### Mutagenesis.

In order to test the role of the key residues H126, D127, E128, R235, and R241 involved in allosteric inhibitor binding and conformational change, various point mutants were prepared and tested for their ability to be stimulated and inhibited by effector molecules in the presence or absence of GFRP. Mutants were quality controlled by sodium dodecyl sulfate polyacrylamide gel electrophoresis and SEC and by using differential scanning fluorimetry (DSF), which demonstrated that the mutated proteins are natively folded and show merely a slightly reduced melting point compared to the wild-type protein (*SI Appendix*, Table S3). Enzyme activity was evaluated using an activity assay measuring the formation of the product H2NTP at 330 nm with increasing GTP concentrations.

With one exception (see below), all discussed GCH1 mutants are still functionally active, but show slightly reduced basal activity compared to wild-type protein (*SI Appendix*, Fig. S3). R241A-GCH1 and R235A-GCH1 lost the sensitivity to BH4 in the absence and presence of GFRP and cannot be inhibited (*SI Appendix*, Fig. S3). This indicates that these residues are essential for binding of BH4 and the adoption of the inhibitory complex, and that the conformational change is essential for the formation of the inhibitory complex. R235A-GCH1 cannot be stimulated by GFRP-Phe (*SI Appendix*, Fig. S3 and Table S4), consistent with the fact that, in the stimulatory complex, the side chain of R235 is involved in a hydrogen bonding interaction with the carbonyl oxygen of L40 of GFRP, which apparently is essential for complex formation.

Finally, the F122A mutant was generated and characterized to check whether the structurally observed, F122-dependent closure of the active site plays a key role in GCH1 function. The F122A mutant is able to form GFRP complexes with BH4 and phenylalanine, elutes as one sharp peak from SEC corresponding to a 350-kDa complex, and shows a sharp melting transition in DSF measurement (*SI Appendix*, Fig. S2*B*). Further, we see an increased thermal stability upon binding of GTP, comparable to wild-type protein (*SI Appendix*, Table S3). This indicates that the protein is properly folded and able to bind substrate and GFRP. Therefore, it was surprising that F122A is completely inactive in enzymatic assays, indicating the importance of F122 in the regulatory mechanism (*SI Appendix*, Fig. S3). Surprisingly, the substrate affinity of the F122 mutant is not changed compared to wild type. The fitted dissociation constant *K*_D_ of GCH1 for the nonhydrolyzable GTP analog 7-deaza-GTP was determined by protein-detected NMR titrations. The two strongest shifting cross-peaks in ^1^H,^13^C HMQC spectra were analyzed and result in an average *K*_D_ of 798 µM. For the F122A mutant, a similar *K*_D_ of 669 µM was determined by fitting the corresponding cross-peaks (*SI Appendix*, Fig. S4). Therefore, the missing enzyme activity of the F122 mutant cannot be explained by reduced affinity to the substrate and must have other underlying causes.

The *K*_D_ in the presence of BH4 could not be analyzed by protein-detected NMR due to severe line broadening of cross-peaks of interacting residues, which experience chemical shift perturbation upon 7-deaza-GTP binding.

### Binding of BH4 Induces Accelerated GTP Binding Kinetics.

**Fig. 6.**
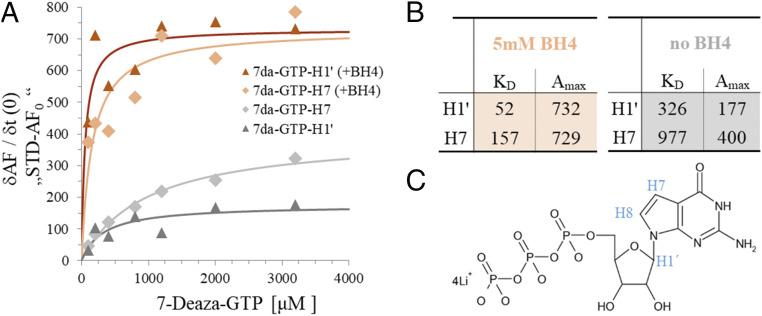
*K*_D_ determination using initial slopes from STD-NMR build-up curves. (*A*) For each ligand concentration, a set of measurements was recorded applying different saturation times (t_sat_). To obtain STD-AF_max_ values and the saturation rate constants k_sat_ from STD-NMR build-up curves, experimental STD-AF values were fit to a rising exponential: STD-AF = STD-AF_max_ (1−exp (−k_sat_ t_sat_)). From the fitting parameters, initial build-up slopes (t_sat_ = 0) were calculated: STD-AF_0_ = STD-AF_max_ k_sat_. These STD-AF_0_ values were plotted against the ligand concentration, yielding a binding isotherm from which *K*_D_ can be derived by fitting the Langmuir binding model: STD-AF_0_ = A_max_/(1+(*K*_D_/c)). A_max_ is a scaling factor representing the maximum STD amplification. Two different 7-deaza-GTP protons (H1′ and H7) were monitored in these experiments. (*B*) All *K*_D_ and A_max_ values are summarized in the table. (*C*) Assignment of relevant protons on structural formula of 7-deaza-GTP.

To shed more light onto the mechanism of BH4-induced inhibition of GCH1, we used saturation transfer difference (STD) NMR spectroscopy to study binding of the substrate analog 7-deaza-GTP to GCH1. STD-NMR can be used to determine the *K*_D_ of ligands (feasible in the range between 10^−2^ and 10^−6^ M) and allows the making of qualitative statements about the change of ligand binding kinetics (28–31). STD-NMR experiments were based on a titration of 7-deaza-GTP to GCH1 in the presence or absence of BH4. We measured a series of STD build-up curves (i.e., STD effects at a constant ligand concentration but increasing saturation times) at each 7-deaza-GTP concentration for the protons H7 and H1′ of the substrate analog. From the concentration dependence of the initial build-up slopes, the dissociation constant as well as the maximum STD amplification for the monitored proton signal can be derived (Fig. 6 *A* and *B*).

The binding isotherms derived from the STD experiments resulted in a comparable affinity of GCH1 to 7-deaza-GTP as determined by protein NMR (*SI Appendix*, Fig. S4) and showed that the BH4-bound, inhibited GCH1 is able to bind substrate in a similar affinity range to the active form of the enzyme. In fact, paradoxically, the affinity of the substrate analog appears to be about sixfold higher for the inhibited enzyme. We concluded that BH4-induced inhibition of GCH1 must be triggered by another, non-affinity-driven mechanism.

Determination of the maximum STD amplification (A_max_) for 7-deaza-GTP (Fig. 6 *A* and *B*) showed an increase by a factor of about 3 for BH4-inhibited GCH1 compared to the uninhibited enzyme. This enhanced STD response is indicative of accelerated ligand binding kinetics, if the effect of changes in the proton network in the environment of the ligand binding site can be excluded (32). We thus conclude that highly accelerated substrate binding kinetics in GCH1 are induced by BH4 binding and thereby could cause the inhibition of GCH1 in the presence of BH4.

## Discussion

We describe here a comprehensive structural study of allosteric regulation of GCH1 and determine two structures of human GCH1-GRFP complexes by cryo-EM. One structure comprises the substrate-bound, allosterically stimulated form of the GCH1−GFRP complex, and the other comprises the allosterically inhibited, substrate-free form. Further, we describe a series of crystallographically determined structures of basally active, nonregulated, and allosterically inhibited GCH1. All structures combined show different aspects of allosteric regulation in unprecedented complexity and detail.

### Proposed Model of Allosteric Inhibition.

From the numerous structures and different functional and biophysical experiments, a model for the allosteric regulation of GCH1 can be developed. To enable the binding of BH4 and coordinate the allosteric inhibitor at the interface of two monomers, the residues R235 and R241 change position and trigger large movements in region C, which include displacement of the central α-helix and change in β-barrel curvature. These rearrangements in a concerted fashion first lead to the shrinking of the central five-helix bundle of the functional oligomer and to a reduction of the β-barrel radius. Secondly, they induce conformational changes in the neighboring subunit, which include displacement of H126 and E128 and formation of a salt bridge between D127 and R235 and, thereby, repositioning of D127. This altered conformation in residues 126 to 128 changes the dynamics of the N-terminally preceding residues 115 to 125. Loop 115–130 (region A) is the only direct connection between the allosteric and the active site and therefore functions as a transmitter of the allosteric signal. In the experimental structures, it transitions from a tight, stable, and closed conformation for the substrate-bound stimulated GCH1, via a more flexible yet conformationally restrained state in active, substrate-free GCH1, to a state of enhanced flexibility triggered by BH4 binding.

Thus two distinct but concerted structural rearrangements occur upon inhibitor binding to the allosteric site, which lead to 1) increased rigidity and decreased mobility in the protein center and 2) enhanced flexibility at the peripheral region A in proximity to the active site. The loosening of the loop structure results in a protein structure that is held in a conformational state that exhibits a distinctly more accessible active site compared to the active state. The absence of any catalytic activity of GCH1 mutant F122A provides further evidence that the nature of the side chain of residue F122, the F122 loop, and region A play a major role in determining enzyme activity.

Our data therefore extend the more simplistic postulate by Maita et al. (18), who proposed a transition of a closed, active to an open, inactive conformation upon allosteric inhibition. In contrast, we unambiguously show that disorder−order transitions and changes in the degree of flexibility of the signal-transducing structural element of region A occur, which differentiate active from inactive GCH1. Further, Maita et al. proposed steric hindrance of substrate binding to be the ultimate cause for the loss of enzymatic activity, while we could clearly show that substrate binding is not at all compromised, and the binding site geometry is unchanged between active and inhibited states.

### Dissociation Rate Controlled Allosteric Inhibition of GCH1.

The observed structural features are strongly reminiscent of the classical models of Monod et al. (33) and Koshland et al. (34) of allosteric regulation. These models describe the transition from a rigid, tense (T), less active state with low affinity for the ligand to a mobile, relaxed (R) high-affinity state (35). More recently, based on experimental data on structure, protein dynamics, and thermodynamics, more complex descriptions for allostery have been developed (36, 37). The largely qualitative, static images of end point protein structures have been replaced by more quantitative, dynamic views of allostery, which, unlike static structural models, are more difficult to describe. Allosteric mechanisms can include intrinsic disorder and local unfolding (38) and remodeling of the energy landscape or can work without any conformational change (39).

We show here that, in allosterically inhibited GCH1, substrate binding is not sterically hindered but occurs with similar affinities to the active states of the enzyme. This finding suggests a mechanism of noncompetitive inhibition for GCH1. However, since we do not observe any structural differences of residues relevant for substrate turnover between active and inactive GCH1, and, in fact, all catalytically relevant residues are identical in position and poised for substrate conversion, it remains unclear why substrate would not be turned over by the inhibited state.

We conclude that BH4-induced inhibition of GCH1 must be triggered by another, non-affinity-driven mechanism and propose here a mechanism of allosteric regulation that involves accelerated substrate binding kinetics. In this model, inhibition is caused by “dissociation before turnover” which, in turn, requires that the residence time of substrate in the active site of the enzyme is shorter than the time required for initiation of substrate conversion.

GCH1 is a very tardy enzyme, and conversion of GTP to H2NTP proceeds extremely slow. Turnover rates were described for GCH1 from bacterial species and are in the range of 0.05 s^−1^ per subunit (40). The initiating step of purine ring hydrolysis to the first reaction intermediate, 2,5-diamino-5-ribofuranosylamino-4(3H)-pyrimidinone-triphosphate (I1), commences at higher turnover rates of 5 s^−1^, while the catalytic steps that occur later, Amadori rearrangement and dihydropyrazine ring closure, are rate limiting. Such slow turnover requires a minimum residence time of substrate in the active site of >0.2 s for formate and I1 formation and >20 s for product formation, in order to provide sufficient time for catalysis to occur. Using a functional ^1^H-NMR based assay that detects formate as the first reaction product of GCH1, we show that allosteric inhibition of GCH1 leads to complete abolishment of enzyme activity including the fast initial purine ring hydrolysis (*SI Appendix*, Fig. S6), suggesting that the relevant parameter for inhibition is the substrate residence time required for this conversion.

Michaelis constants for mammalian GCH1 have been determined to be in the two-digit micromolar range (17). Using the substrate analog 7-deaza-GTP, we determined *K*_D_ of ∼100 and 600 µM for BH4-bound inhibited and free, active hGCH1. The slightly higher values for 7-deaza-GTP may be explained by the difference in molecular structure, that is, the replacement of the N7 atom by a carbon atom.

Assuming a substrate binding affinity of 100 µM and diffusion-limited association rates (10^6^ M^−1^⋅s^−1^) an unrealistically high dissociation rate constant of 100 s^−1^ would be required, way too fast compared to the 5 s^−1^ rate required for purine ring hydrolysis. Therefore, it is more likely that substrate association occurs on a slower timescale (∼10^4^ M^−1^⋅s^−1^) by assuming that GTP folds slowly into the active site pocket of catalytically active GCH1 after rapid electrostatically driven triphosphate binding. Dissociation would then occur in the ∼1 s^−1^ timescale. These slower binding kinetics would roughly correspond to the above described target range of residence times of ∼1 s needed for the substrate conversion.

Here, we provided evidence for enhanced substrate dissociation rates in allosterically inhibited GCH1 using STD-NMR data, which show a threefold STD response enhancement (Amax increase) for the allosterically inhibited form of GCH1 corresponding to an increase in dissociation rate constants by factors between 10- and 100-fold (32). In light of the required residence time of substrate in the GCH1 active site, such an acceleration of substrate dissociation could thus lead to a switch between active and completely inactive states by reduction of the substrate residence time from ∼1 s to between ∼0.01 and ∼0.1 s.

We have further shown that the substrate analog 7-deaza-GTP binds, in fact, slightly more tightly to the allosterically inhibited GCH1 (factor of ∼6). In order to satisfy thermodynamics, assuming acceleration of dissociation by factors of 10 to 100 requires that the corresponding association rates must increase by this factor multiplied by the increase in binding affinity, as such 60- to 600-fold.

These effects on association and dissociation rates can be plausibly explained by our structural findings. Allosteric inhibition by BH4 leads to enhanced flexibility of region A, the F122-containing loop. The loop structure acts as a lid above the active site, which effectively traps GTP during its turnover and shields the reaction chamber from bulk solvent (18). None of the residues in region A are directly involved in the catalysis or recognition of substrate. A change in the intrinsic flexibility of this lid region should have direct influence on substrate binding kinetics. The enhanced flexibility observed in the allosterically inhibited state will enable a faster formation of the GTP bound state of GCH1 and result in faster association rates. Likewise, faster dissociation rates are triggered by the higher tendency of the F122 loop to open up due to the less rigid attachment to neighboring amino acid stretches. Thus, the interpretation of our structural data is consistent with the hypothesis that a strong acceleration of the association of substrate overcompensates the acceleration of the dissociation of substrate, leading to the observed inverted binding affinities.

The absence of activity of the F122A mutant may, in part, be explained by a similar mechanism. While we would not expect the mutant to trigger the same quaternary structural rearrangements observed for the allosterically inhibited enzyme, absence of the bulky aromatic side chain at the center of the lid loop will have similar effects on the accessibility of the active site and substrate binding kinetics, explaining the inability of this point mutant of GCH1 to generate product, while its substrate binding affinity is identical to the wild-type enzyme as shown here by ^1^H,^13^C HMQC NMR.

While negative allosteric modulators are generally believed to shift the equilibrium distribution of enzyme conformations to favor a catalytically incompetent structure, the kinetics of conformational exchange or substrate binding is barely addressed in the literature. For IMP dehydrogenase, kinetic control of allosteric activation was described (41). In this case, the positive control functions via a ≥65-fold increase of k_cat_ in the presence of the effector molecule K^+^. Riera et al. (41) use the term “kinetic control” to describe a change in enzyme kinetics and not for a regulation of the enzyme via altering the binding kinetics as it is used in this study. Allosteric regulation by binding kinetic control as proposed here, triggered by an increase in the k_off_ and driving the substrate residence time below the threshold of the turnover rate, is so far not described in literature.

### GFRP Acts as a Scaffolding Protein that Sensitizes GCH1 to React to Physiological Levels of Effector Molecules BH4 and Phenylalanine.

It is very likely that the role of GFRP is limited to that of a scaffolding protein. It stabilizes the active or inactive conformations of the GCH1 decamer by direct GFRP−GCH1 interactions and by enhancing the binding affinity of allosteric inhibitors. This notion fits well with the relatively small effects of GFPR on enzyme kinetics and the conformational rearrangements. GCH1 alone can be fully inhibited by allosteric inhibitors. In the presence of GFRP, the K_i_ of allosteric inhibitors is decreased by a factor of 10 (16). Comparison of the inhibitory GCH1−GFRP−BH4 complex with allosterically inhibited GCH1 shows that binding of GFRP does not change the overall conformation of GCH1 significantly (Fig. 4*G*). Concerning GCH1 stimulation by GFRP, from enzyme kinetics, we know that GFRP-Phe merely reduces the positive cooperativity of GCH1 and, as a result, slightly stimulates the enzyme’s activity in the presence of subsaturating concentrations of GTP without an effect on V_max_ (17, 20). Again, conformational changes of the catalytically active GCH1 decamer between active GCH1 and the stimulatory GCH1−GFRP-Phe complex are small. GFRP seems to help stabilize the active conformation in each of the 10 individual active sites of GCH1, thereby reducing the cooperativity between the active sites and allowing for independent binding of substrate. Although the effects of GFRP on the GCH1 activity appear to be small in terms of enzyme kinetics and structural rearrangements, it has dramatic effects on the response of GCH1 to physiological concentrations of effector molecules BH4 and phenylalanine as originally described by Harada et al. (17).

In conclusion, this comprehensive structural and mechanistic study shows GCH1 in an ensemble of ligand-induced states from active to inactive and thereby provides insights into the mechanism of allosteric regulation of GCH1 in unprecedented detail. Since the BH4 pathway is currently perceived as an attractive target to treat pain disorders (6, 42, 43), with GCH1 being the target with human genetics validation, the understanding of the details of allosteric GCH1 inhibition as well as the methods used here will prove highly useful to identify potential drug candidates that selectively modulate its activity.

## Methods

### Protein Expression and Purification.

The hGCH1 (42-250, N-terminal 6xHis Tag and TEV cleavage site) and hGFRP (1-84, N-terminal 6xHis Tag and TEV cleavage site) constructs were cloned into pET17b or pET28s, respectively, and transformed into BL21(DE3) *E. coli *cells. Cells were grown in lysogeny broth media until the optical density (OD) (A600) reached 0.6. The cultures were induced with 1 mM Isopropyl β-D-1-thiogalactopyranoside (IPTG) and grown for an additional 16 h at 20 C°. The cells were pelleted by centrifugation. For purification, the bacterial cell pellet was thawed, suspended in lysis buffer (25 mM Tris⋅HCl, pH 7.4, 300 mM NaCl, 1 mM dithiothreitol, complete protease inhibitor mixture [Roche]), and lysed by sonication. Crude cell lysate was clarified by centrifugation at 45,000 × *g* for 45 min. Supernatant containing 6xHis-tagged protein was purified over Ni-NTA agarose column (Protino Ni-NTA Agarose, Macherey-Nagel). The 6xHis Tag of hGFRP was cleaved using TEV protease. Both proteins were further purified via SEC (Superdex 200 Increase 10/300 GL) using 150 mM NaCl, 25 mM Tris⋅HCl pH 7.4. The protein-containing fractions were pooled and concentrated to 7 mg/mL using a centrifugal filtering device (Millipore, 30-kDa molecular weight cutoffs).

### Protein Crystallization.

For crystallization, the protein buffer was exchanged by SEC (Superdex 200 increase 10/300 GL, GE Healthcare) using 100 mM sodium phosphate buffer pH 5.5 and concentrated up to 6 mg/mL using Amicon centrifugal filters (50-kDa cutoff). All crystals were obtained by sitting drop vapor diffusion using 96-well three-drop SWISSCI plates (MolecularDimensions). The protein was mixed 1:1 (300 + 300 nL) with reservoir solution and was equilibrated against the reservoir. All crystallization trials were set up using the Mosquito pipetting robot system (TTP-labtech).

GCH1 apo crystals were obtained from a reservoir solution containing 1.26 M ammonium sulfate, 0.1 M Tris pH 8.5, and 0.2 M lithium sulfate. For the complexation with small molecules, 1 mM 7-deaza-GTP (TriLink; N-1044-10), 0.1 mM 8-oxo-GTP (Jena Bioscience NU-1116S), or 1 mM AXSP0056BS (cpd-1) was added to GCH1 (6 mg/mL) prior to crystallization. GCH1 7-deaza-GTP cocrystals were obtained from a reservoir solution containing 0.1 M Morpheus buffer 1, 30% Morpheus ethylene glycol and PEG 8000 mixture (EDO_P8K) and Morpheus ethylene glycols (Morpheus HT-96; E10), and GCH1 AXSP0056BS cocrystals from 0.2 M magnesium chloride, PEG8000; Tris pH 7.0 or 30% GOL-P4K, 0.1 M Morpheus amino acids, 0.1 M Morpheus buffer 2 pH 7.5 (Morpheus HT-96; G7) (asymmetric, partial occupied crystal form). Crystals grew at 20 °C after 2 d to 10 d. All crystals, except those grown in Morpheus screens, were flash frozen in liquid nitrogen and cryoprotected using 28% Glycerol.

### X-ray Data Collection, Processing, and Refinement.

X-ray diffraction data were collected at the Swiss Light Source at the PXIII and PXI beamline, and processed with the autoPROC pipeline (44) using the XDS package (45). Resolution cutoffs were calculated using STARANISO (46). Data processing statistics are listed in *SI Appendix*, Table S1. The model of GCH1 was manually built using Coot (47), and the resulting model was improved by iterative rounds of manual rebuilding and refinement with autoBuster (48). The phases were obtained by molecular replacement [Phaser-MR (49)] using the hGCH1 structure (1FB1) as search model. The crystals contained 5 to 20 GCH1 monomers per asymmetric unit. The final models of all crystal structures and the structure factors have been deposited in the Protein Data Bank (PDB) (PDB ID codes 6Z86, 6Z87, 6Z88, and 6Z89).

### Sample Preparation for EM.

For complex formation, hGCH1 and hGFRP were mixed using a 1.3× hGFRP excess. The mixed proteins were diluted by 1:10 in complex buffer and incubated for 30 min at 4 °C. The identification of suitable buffer conditions was key to the preparation of high-quality grids. The final puffer composition was identified using the ProteoPlex technology (50). One hundred millimolars sodium phosphate pH 5.5, 80 mM NaCl, and 20 mM phenylalanine were used to form the stimulatory complex, and 100 mM sodium citrate pH 5.75 and 0.1 mM BH4 (Sigma Aldrich; T4425) were used for the formation of the inhibitory complex. The two distinct protein complexes were purified using a Superose 6 Increase 10/300 GL column and the respective complex buffers. The respective complex fractions were pooled and concentrated up to 1 mg/mL to 1.6 mg/mL. The purified samples were never kept at 4 °C longer than 48 h prior to grid preparation.

Shortly before grid preparation, the stimulatory complex was diluted with Phe containing buffers to 0.6 mg/mL so that the final buffer composition consisted of 50 mM sodium phosphate pH 5.5, 20 mM NaCl, and 20 mM Phe. Furthermore, 0.1 mM 8-oxo-GTP (Jena Bioscience; NU-1116) was added. The inhibitory complex was diluted to 0.25 mg/mL, and 40 mM sodium citrate pH 5.75 and 0.1 mM BH4. 3 µL of protein solution were applied to freshly glow-discharged C-flat CF-1.2/1.3-4C grids and plunge frozen in liquid ethane using a Vitrobot (Thermo Fisher).

### Data Acquisition and Processing of EM Datasets.

Micrographs were automatically recorded using EPU on a Titan Krios microscope (Thermo Fisher) operated at 300 kV equipped with a K2 direct electron detector in electron counting mode at a nominal magnification of 130,000×, corresponding to a calibrated pixel size of 1.077 Å. Dose fractionated 8-s movies of 40 frames were recorded with a total electron dose of 55 e/Å^2^ using defocus values of 1.0 μm to 2.4 µm.

A total of 2,698 micrographs was collected for the inhibitory dataset, and 3,121 images were collected for the stimulatory complex. Whole-image drift correction of each movie was performed using MotionCorr (51). The contrast transfer function (CTF) was determined using CTFFIND4 (52) in the RELION 3.0 workflow (53). Initially ∼40,000 particles were manually picked with EMAN boxer (54) and subjected to two-dimensional (2D) reference-free classification in RELION (53) to check the quality of the particle images and generate 2D class averages for autopicking in RELION. Approximately 350,000 to 750,000 particles with a box site of 250 × 250 pixels were extracted. Afterward, the dataset was cleaned by 2D and 3D classification. A low-passed filtered volume of the rat GCH1-GRFP crystal structures (1is7; 1wpl) was used as a starting reference for 3D classification. The stimulatory and inhibitory complexes were reconstituted from 122,275 or 254,907 particles, respectively, using RELION 3Dauto-refine. The refined particles were subjected to per-particle CTF refinement and Bayesian polishing in RELION 3.0 (53), yielding maps of 2.9- and 3.0-Å resolution determined by the gold standard 0.143 Fourier shell correlation (FSC) criterion using the postprocessing procedure in RELION 3.0. For both complexes, the same mask was applied. Local resolution was estimated using MonoRes (55).

Structures of hGCH (1fb1), hGFRP (7acc), and rGCH-GFRP (1wpl, 1is7) were docked into our final cryo-EM maps using Chimera (56). Docking of the individual subunits was improved by rigid body fitting in Coot. Model adjustment and refinement were performed iteratively in Coot (47) and Phenix (57), and the statistics were examined using Molprobity (58) until no further improvements were observed. The final model was also evaluated using FSC analysis against the map and using EMRinger (59) to compare the fit of the model backbone into the cryo-EM map. The model statistics showed good geometry and matched the cryo-EM reconstruction (*SI Appendix*, Table S2). The structures of the hGCH−hGFRP inhibitory and stimulatory complexes have been deposited in PDB (PDB ID codes 6Z80 and 6Z85). The respective cryo-EM density maps have been deposited in the Electron Microscopy Data Bank (accession codes EMD-11113 and EMD-11114)

### Enzyme Kinetics.

GTPCH-I specific enzyme activity was determined by spectrometric measuring of the concentration of its direct product H2NTP. Synergy H1 (BioTek Instruments) and Gen5 2.01 software were used to evaluate H2NTP concentration and the maximal turnover rate (*V*_max_) within each measuring interval. H2NTP concentration was measured at 330 nm over a measuring period of 2 h using a measuring interval of 2 min to 4 min at 37 °C. Samples were prepared using 2 μM GTPCH-I, varying concentrations of GTP (1 μM to 2,000 µM) and in the absence or presence of 15 mM phenylalanine, 0.1 mM BH4, or 3 μM GFRP. The assay buffer used was 50 mM Tris/HCl, pH 7.5, 100 mM KCl. The specific enzyme activity (A) was calculated using the path length of 0.15 cm and the extinction coefficient ε300 nm = 6,300 M^−1^⋅cm^−1^ H2NTP. The resulting specific activity values were plotted against increasing substrate concentration ([S]) and fitted by means of Origin software using the Hill equation, which was additionally used to determine the final *V*_max_ values, Hill coefficients (*n*) and Michaelis−Menten constants (*K*_m_). Statistical analysis was performed using Welch’s *t* test. All enzymatic data were, at least, measured in triplicate (*n* = 3).

### STD-NMR Titration Assay.

All NMR experiments were performed at 25 °C on a Bruker HD 600-MHz spectrometer equipped with a cryogenic QCI probe head. Deuterium oxide (D_2_O) NMR buffer solution containing 40 mM of a NaH_2_PO_4_ and Na_2_HPO_4_ mixture with 150 mM sodium chloride (NaCl) was adjusted to a pD of 7.4 (corrected). Suppression of broad protein resonances was achieved by a T1ρ filter (60), formed by a 50-ms spin lock pulse. STD-NMR spectra were acquired with WATERGATE water suppression using W5 hard pulse trains (61). Difference spectrum ligand signal intensities were divided by corresponding off-resonance spectrum signal intensities, resulting in relative STD intensity values. Multiplication by ligand excess factor yielded STD amplification factor (STD-AF) values (28). For selective protein signal saturation, a cascade of Gaussian-shaped pulses was applied. Series of measurements with different total saturation times (t_sat_) covering a range between 1.2 and 7.2 s were recorded for extraction of initial slopes from STD-AF build-up plots. An additional relaxation delay varying from 2.8 s to 0.4 s was inserted before the saturation cascade to keep constant the total time per scan. On-resonance irradiation was set to 0.16 parts per million (ppm), and off-resonance irradiation was set to 66 ppm. Depending on the ligand concentration, 64 to 1,024 scans were acquired for the STD experiments (32 to 512 scans each for on- and off-resonance). Considering the STD-AF to be directly proportional to the fraction of bound receptor gives rise to a hyperbolic dose–response curve (specific one-site binding model) when STD-AF values are measured with different ligand concentrations (31). Apparent (saturation time dependent) dissociation constants for 7-deaza-GTP were calculated by plotting all STD-AF values of a single saturation time (e.g., 3.6 s) against the ligand concentration and fitting the data points to the Langmuir binding model (62). To overcome the saturation time dependency, additionally, extrapolated *K*_D_ values were calculated from initial slope values (STD-AF_0_) of the STD-AF build-up (63). Fast protein−ligand rebinding during t_sat_ and accumulation of saturated ligand molecules yields lower STD-AF values predominantly for high ligand-to-protein excess factors, and this results in higher apparent *K*_D_ values. These effects are minimized as t_sat_ approaches 0.

### NMR Titrations and Production of Isotope-Labeled Protein.

Perdeuterated protein with selectively labeled protonated and ^13^C-labeled alanine, isoleucine, leucine, and valine methyl groups {Ala^β^-[^13^CH_3_]; Ile^δ1^-[^13^CH_3_]; Leu^δ^,Val^γ^-[^12^CD_3_/^13^CH_3_]} was expressed as described in the literature (64). Purification was done as described for the unlabeled protein. All spectra were recorded on a Bruker Avance III 800 MHz NMR spectrometer equipped with a cryogenic triple-resonance probe at 298 K. Fifty micromolars perdeuterated GCH1 monomer with selectively labeled methyl groups was dissolved in 1× PBS in D2O. The ^1^H,^13^C-HMQC spectra of the protein were recorded with increasing concentration of the nonhydrolyzable GTP analog 7-deaza-GTP up to a final concentration of 3.5 mM. Spectra were processed with Bruker Topspin 3.5 and analyzed using CcpNmr (65) analysis version 2. Chemical shift perturbation (CSP) was calculated using Eq. **1** and the binding curve was fitted using Eq. **2**.

$$\text{CSP}=\sqrt{{\left(\delta_{H}-\delta_{Href}\right)}^{2}+\frac{{\left(\delta_{N}-\delta_{Nref}\right)}^{2}}{100}},$$ [1]

where *δ* is the chemical shift of the spectrum in the apo (*ref*) or in the presence of 7-deaza-GTP in the proton (*H*) and nitrogen (*N*) dimension.

$$y=\frac{{\text{CSP}}_{max}\cdot \left(x+K_{\text{D}}+\left[P\right]-\sqrt{{\left(x+K_{\text{D}}+\left[P\right]\right)}^{2}-4\cdot x\cdot \left[P\right]}\right)}{2\cdot \left[P\right]},$$ [2]

where CSP_*max*_ is the fitted chemical shift of the saturated complex and [*P*] is the GCH1 concentration.

### DSF.

The Tm of proteins and protein complexes was determined by DSF using the Thermofluor Bio-Rad CFX384 and the Bio-Rad CFX Manager software. Ten micromolar protein solution was equipped with 5× SYPRO Orange Protein Gel Stain (Invitrogen) reaching a total volume of 10 μL. Effector molecules and GFRP were added in different concentrations to test their influence on thermal stability; 384 well plates were heated up using a temperature ramp from 15 °C to 95 °C in 1 °C/min steps.

### Analytical Size Exclusion Chromatography.

The respective proteins were mixed in equimolar quantities and incubated for 10 min at 4 °C in 100 mM sodium citrate pH 5.75 and in the presence or absence of the respective effector molecules phenylalanine (15 mM) or BH4 (0.2 mM). Ten to forty micrograms of protein was injected into an analytical SEC column, Superdex 200 Increase 5/150 GL (GE Healthcare), using an ÄKTAmicro system. The system was run at 0.2 mL/min, and the mobile phase consisted of 100 mM sodium citrate pH 5.75 and respective effector molecules. The elution of hGCH1 or hGCH1−hGFRP complexes was detected via absorbance measurements at 280 nm using an ultraviolet absorbance detector. Comparative analysis of retention times of wild-type GCH1−GFRP in the absence and presence of effector molecules as well as single-component injections were used to determine successful complex formation of mutated GCH1.

### Functional Assay with NMR Detection.

For the functional assay, each NMR sample was prepared with 10 μM GCH1 and 170 μM or 600 μM test compound. The PBS NMR buffer additionally contained 300 μM GTP (Cayman Chemical cat. no. 16060). After a reaction time of 45 min (25 °C), a certain amount of substrate molecules (GTP) has been converted by GCH1 into 7,8-Dihydroneopterin (H2NPT) and the by-product formate (deprotonated formic acid). For product detection, a WATERGATE 1H-NMR spectrum was recorded (128 scans). We used the integrated NMR signal of the formate α-proton at 8.33 ppm as a relative enzyme activity readout. The formate integral of a negative control sample (dimethyl sulfoxide) has been set to 100%. An inhibitory active test compound decreases the GTP turnover and leads to a reduced relative formate signal integral (% inhibition). DAHP was used as a positive control.

## Supplementary Material

Supplementary File

## Data Availability

All study data are included in the article and *SI Appendix*. The final models of all crystal structures and the structure factors have been deposited in PDB, http://www.wwpdb.org (PDB ID codes 6Z86, 6Z87, 6Z88, and 6Z89).
