# Supplementary material for: A hybrid approach reveals the allosteric regulation of GTP cyclohydrolase I
Source: Proc Natl Acad Sci U S A. 2020 Nov 23;117(50):31838–49. doi: 10.1073/pnas.2013473117 (PMC7750480; doi:10.1073/pnas.2013473117)
Supplement: Supplementary File [file pnas.2013473117.sapp.pdf]

Supplemental Material

Supplemental Table 1: Data collection and refinement statistics for hGCH1 co-crystallized in complex with AXSP0056BS or 7-deaza-GTP**.** Values in parentheses represent higher resolution shell; values without parentheses refer to the total resolution range.

**Experimental data**				
PDB ID	6Z86	6Z88	6Z89	6Z87
Protein species	hGCH1	hGCH1	hGCH1	hGCH1
Experimental setup	co-crystal 7-deaza-GTP	co-crystal AXSP0056BS	co-crystal AXSP0056BS (partially occupied)	apo
**Data Collection**				
Wavelength (Å)	0.99988	0.99986	0.99987	0.99986
Space group	P1	C2221	P6522	P6522
Unit cell parameters				
a, b, c (Å)	87.9 88.6 163.6	112.4 161.5 271.7	121.8 121.8 357.2	109.9 109.9 387.2
α, β, γ (°)	85.2 88.9 83.5	90.0 90.0 90.0	90.0 90.0 120.0	90.0 90.0 120.0
Resolution (Å)	87.739-2.206 (2.457-2.206)	135.821-2.687 (2.971-2.687)	105.470-2.366 (2.634-2.366)	95.156-2.904
Observed reflections	257298 (13019)	483392 (20327)	1197104 (56604)	506777 (56604)
Unique reflections	142765 (7139)	47926 (2396)	44678 (2235)	44678 (21546)
Completeness (spherical) (%)	58.1 (10.5)	69.4 (13.5)	68.9 (12.8)	59.8 (9.8)
Completeness (ellipsoidal) (%)	89.7 (62.4)	93.9 (58.2)	96.1 (78.8)	93.6 (77.2)
Redundancy	1.8 (1.8)	10.1 (8.5)	26.8 (25.3)	18.6 (15.8)
R_{merge} (I)	0.045 (0.337)	0.201 (1.506)	0.109 (2.593)	0.309 (2.801)
R_{pim} (I)	0.045 (0.337)	0.066 (0.541)	0.021 (0.519)	0.073 (0.711)
I/σ (I)	6.2 (1.5)	8.9 (1.5)	24.8 (1.6)	13.7 (1.9)
$CC_{1/2}$	0.998 (0.772)	0.998 (0.530)	1.000 (0.686)	0.997 (0.670)
**Refinement**				
Resolution (Å)	87.7-2.2	135.8-2.6	46.3-2.4	95.2-2.6
R_{work} (%)	18.10	21.3	22.8	22.8
R_{free} (%)	20.80	24.9	23.4	25.5
rmsd bond length (Å)	0.009	0.008	0.009	0.007
rmsd bond angles (°)	1.17	1.08	1.15	0.93
Mean/Wilson B (Å^2)	51/49	74/69	80/75	76/75
Ramachandran plot				
Favoured (%)	98.38	97.77	96.75	97.70
Outliers (%)	0.00	0.12	0.38	0.22
Sidechain outliers (%)	3.0	3.9	5.3	1.8

www.pnas.org/cgi/doi/10.1073/pnas.2013473117

Supplemental Table 2: Data collection, processing and refinement statistics for hGCH1-hGFRP inhibitory and stimulatory complex cryo -EM structures.

**Experimental data**		
PDB ID	6Z85	6Z80
ID	Inhibitory complex	Stimulatory complex
Protein	hGCH-hGFRP	hGCH-hGFRP
Ligands	BH4	Phenylalanine<br>8-oxo-GTP
**Data Collection and Processing**		
Microscope	FEI Titan Krios	FEI Titan Krios
Voltage (kV)	300	300
Camera	Gatan K2 Summit	Gatan K2 Summit
Exposure time (s)	8	8
Total Dose ($e^-/Å^2$)	55	55
Dose per frame ($e^-/Å^2$)	1.38	1.38
Defocus range (µm)	1.0-2.5	1.0-2.5
Pixel size (Å) (calibrated)	1.077	1.077
Magnification (nominal)	130,000x	130,000x
Symmetry imposed	D5	D5
Number of micrographs	2688	3121
Initial particle number	1,871,460	1,272,592
Final particle number	560,802	122,310
Map resolution (Å)	2.9	3.0
FSC threshold	0.143	0.143
**Refinement**		
Map-sharpening B factor ($Å^2$)	-128	-104
Model composition		
Protein (residues)	2360	2660
Ligands	10xBH4	10xPhe; 10x8-oxoGTP
RMSD bond length (Å)	0.013	0.010
RMSD bond angles (°)	1.665	1.587
Rotamer outliers (%)	4.85	3.85
ADP (B-factor) (min/max/mean)		
Protein	3.07/25.22/9.14	5.22/30.24/12.07
Ligand	4.54/26.07/5.74	7.16/49.68/9.83
Ramachandran plot		
Favoured (%)	96.93	96.05
Allowed (%)	3.07	3.56
Outliers (%)	0.00	0.38
All-atom clashscore	7	3
MolProbity score	2.47	2.53

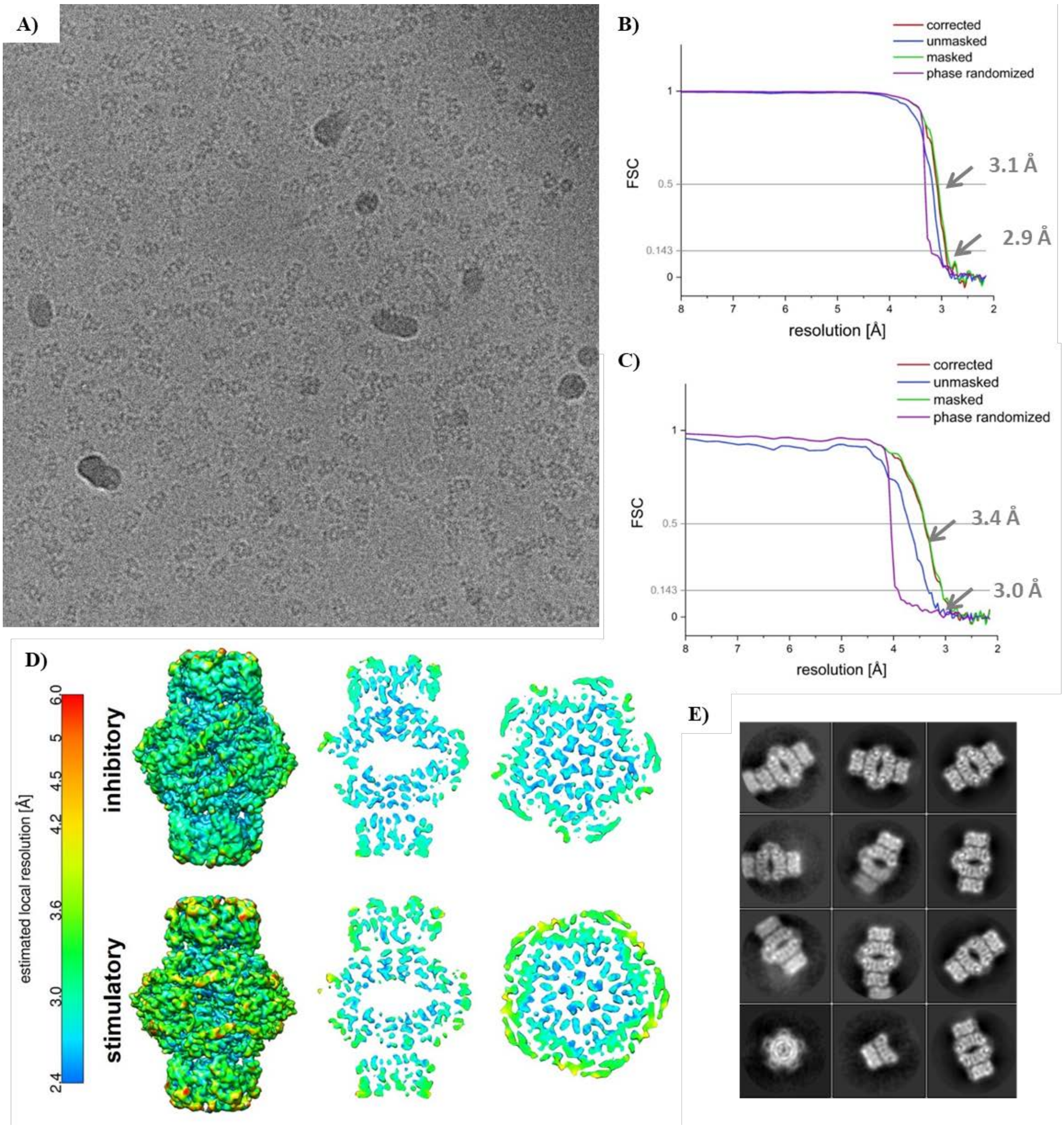

Supplemental Figure 1: Details of EM data collection processing and structure validation. A) Exemplary microscopic image of the inhibitory complex. FSC of the inhibitory **B)** and the stimulatory **C)** complexes between two half-maps as a function of spatial frequency were calculated. FSC is shown for the original, unmasked half-maps (blue), masked half-maps that had most of the membrane and genome density removed (green), and phase-randomized half-maps (purple) in which phases were randomized at frequencies higher than 1/3.5 Å or 1/4 Å. The phase-randomized FSC drops sharply at the cutoff frequency below the noise threshold (0.143), as expected. The phase-randomization test was used to take the effect of masking on the half-maps into account before calculating the final, corrected FSC curve (red). Good agreement between the masked and corrected curves indicated that masking did not cause overestimation of resolution. The corrected curve drops below the noise threshold at 1/2.9 Å and 1/3.0 Å indicating a resolution of 2.9 Å in the reconstruction of the inhibitory complex and 2.9 Å for the stimulatory complex. **D)** Local resolution estimation and FSC functions surface and central slices of the inhibitory (top) and stimulatory (bottom) hGCH1-GFRP complexes colored according to local resolution estimation using MonoRes[55]. **E)** Exemplary 2D Classes of the inhibitory complex generated using Relion.

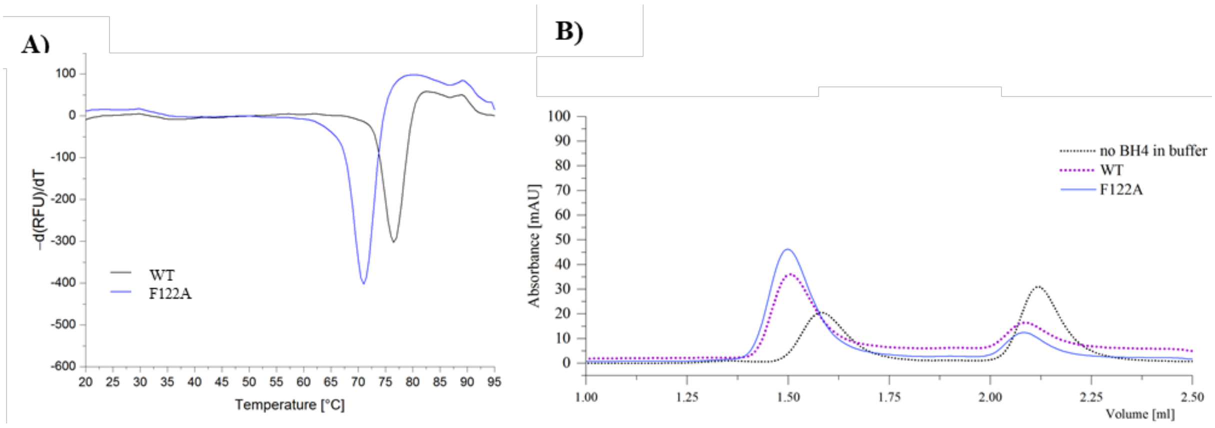

Supplemental Figure 2: Characterization of F122A mutant using DSF and analytical SEC. A) Determination of protein melting points using DSF. T_M of wtGCH1 is 76.5 °C and a T_M of 71.0°C was measured for F122A-GCH1. **B)** 'Analytical size exclusion profile shows that both proteins (wtGCH1 (purple) F122A-GCH1 (blue)) are able to form GFRP complexes in presence of BH4. The black curve shows the mixture of GFRP and wtGCH1 in absence of BH4 in all buffers. According to molecular weight standards, the molecular weight of GCH1-GFRP (retention time: 1.48 ml; M: 257 kDa), GCH1 (retention time: 1.59 ml; M: 170 kDa) and GFRP (retention time: 2.13/2.06 ml; M: 23/30 kDa) was calculated based on their retention times.

Supplemental Table 3: Differential scanning fluorimetry (DSF) measurement TM values of GCH1 and mutated GCH1 variants. The protein melting points were determined in presence and absence of GTP.

protein	TM [°C]	ΔTM WT [°C]	TM + GTP [°C]	ΔTM WT+GTP [°C]
WT	76.5	-	83.2	7.0
F122A	71.0	-5.5	75.2	4.2
R235A	70.7	-5.8	81.2	11.2
D127A	73.5	-3.0	78.0	5.0
E128A	76.0	-0.5	78.5	4.3
H126A	74.2	-2.3	80.0	6.0
R241A	69.3	-7.2	86.2	17.7

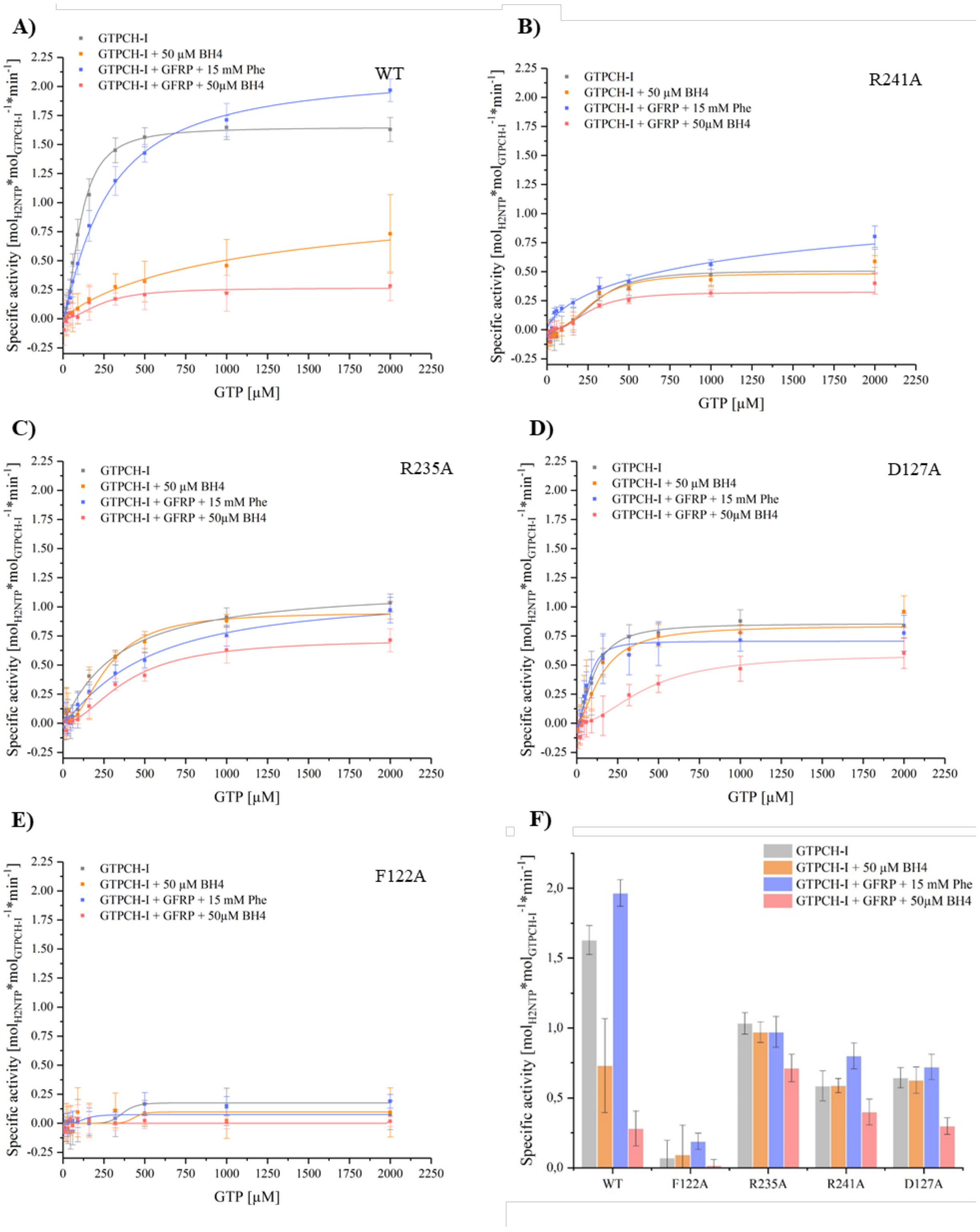

Supplemental Figure 3: Enzyme kinetics of F122 and allosteric site mutants. Specific enzyme activity of **A)** WT, **B)** R241A, **C)** R235A, **D)** D127A and **E)** F122A GCH1 is plotted versus titrated substrate concentrations (1–2000 µM GTP) (n = 3). **F)** Bar-plot of the specific enzyme activity at 2000 µM GTP compares the basal activity of the enzymes (grey), the ability to be stimulated (blue) and the ability to be allosterically inhibited in presence (salmon) and absence (orange) of GRFP. The Graph compares GCH1, F122A-GCH1, R235A-GCH1 and R241A-GCH1.

Supplemental Table 4: Kinetic parameters of GCH1 F122 and allosteric site mutants. Enzymatic data was derived for wt GCH1 without GFRP and distinct mutants (not stimulated/inhibited) spectrometric measuring the concentration of its direct product H2NTP. From this data (shown in supplemental figure 3, grey) values for V_{max} K_M, and Hill coefficient n_H as well as their deviations could be obtained.

	**V_{max} [molH2NTP/(min*molGCH)]**	**σ V_{max}**	**K_M [μM]**	**σ K_M**	n_H	σ n_H
WT	1,65	0,03	105,60	5,91	1.82	0.13
F122A	0,10	0,06	434,29	591,39	14.00	129,49
R235A	1,17	0,11	328,28	67,33	1.09	0.17
D127A	0,72	0,16	483,05	206,98	1.44	0.48
R241A	0,51	0,07	305,98	51,83	2.72	1.18

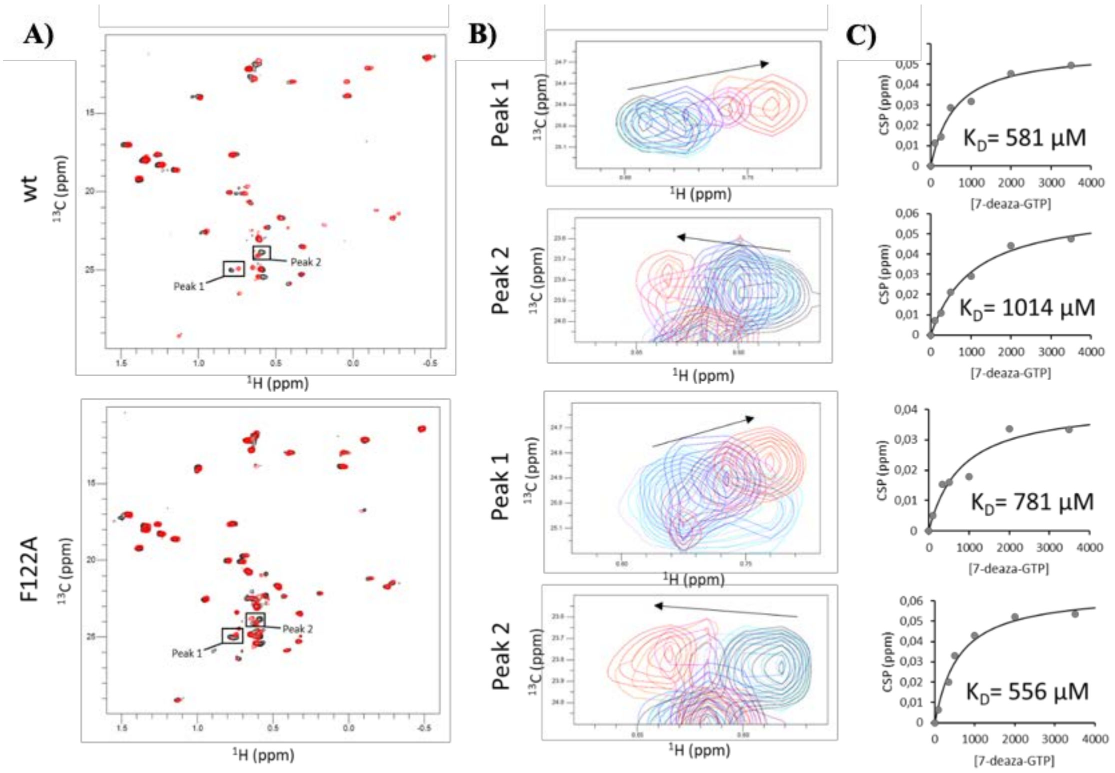

Supplemental Figure 4: Determination of KD of GCH1 for the non-hydrolysable GTP analog 7-deaza-GTP by protein detected NMR titrations. A) ^{1}H,^{13}C correlation spectra of GCH1 wt (upper panel) or F122A (lower panel) free (black) and in presence of 3.5 mM 7-deaza-GTP (red). Boxes mark the peaks used for fitting the titration curves. **B)** Peak 1 and Peak 2 from A in the presence of increasing concentrations of 7-deaza-GTP. **C)** Chemical shift perturbation extracted from spectra in B is plotted against the 7-deaza-GTP concentration (dots). The fitting curve is depicted as black line and the dissociation constant is indicated.

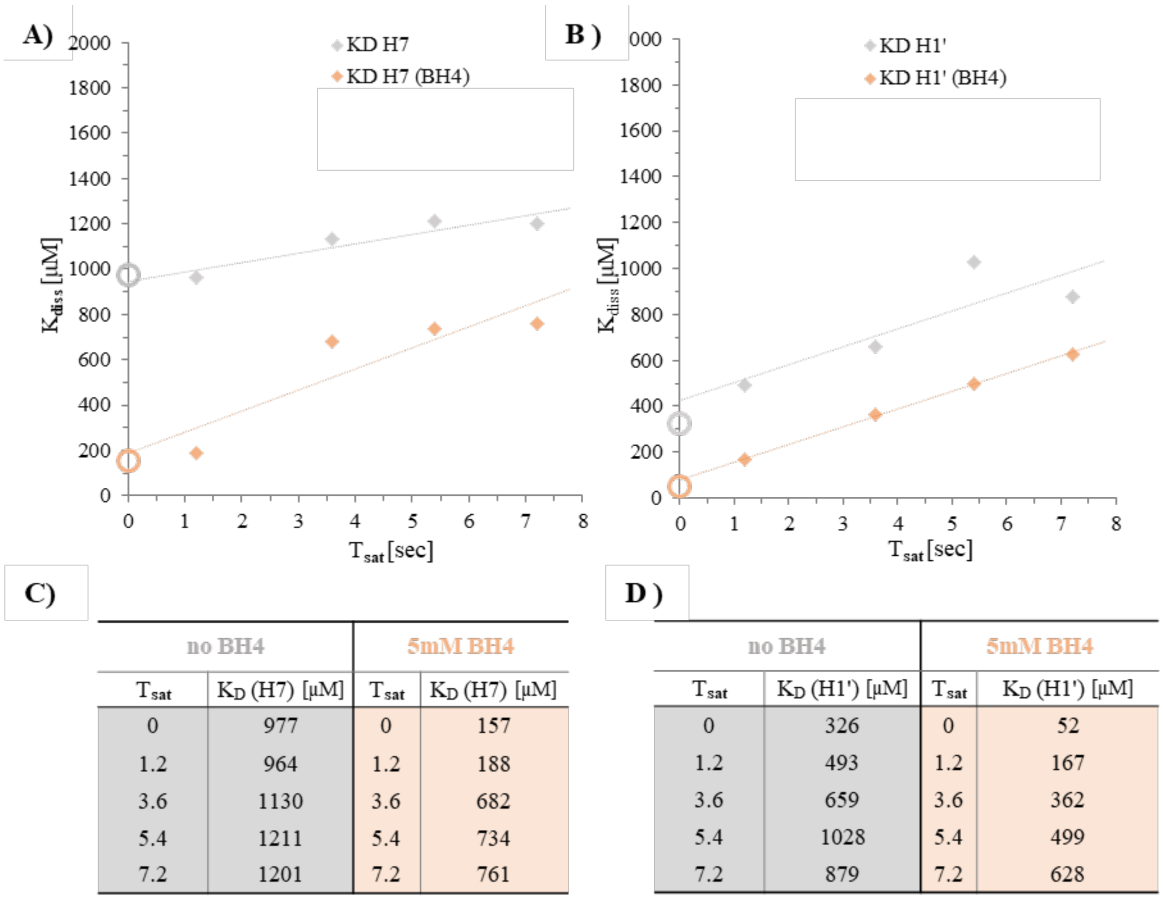

C)

no BH4		5mM BH4	
T_{sat}	K_D (H7) [µM]	T_{sat}	K_D (H7) [µM]
0	977	0	157
1.2	964	1.2	188
3.6	1130	3.6	682
5.4	1211	5.4	734
7.2	1201	7.2	761

D)

no BH4		5mM BH4	
T_{sat}	K_D (H1') [µM]	T_{sat}	K_D (H1') [µM]
0	326	0	52
1.2	493	1.2	167
3.6	659	3.6	362
5.4	1028	5.4	499
7.2	879	7.2	628

Supplemental Figure 5: Comparison of apparent K_D values and extrapolated K_D values derived from STD NMR data by different approaches. Apparent K_D values (diamonds) obtained from measurements with a single saturation time are represented as a function of t_{sat}. Initial slopes from STD-AF build-up plots gave extrapolated K_D values for t_{sat} = 0 (circles) depicted in Figure 6. For longer saturation times the apparent K_Ds tend to higher numeric values leading to an underestimation of the binding affinity. With lower t_{sat} values apparent K_Ds approach the extrapolated K_D for t_{sat} = 0. Dotted lines are added to aid visualization. Two different 7-daza-GTP protons (H1' and H7) were monitored in these experiments. Panel **A)** and **C)** show the results for the H7 proton, while the data for H1' is depicted in panel **B)** and **D)**.

.

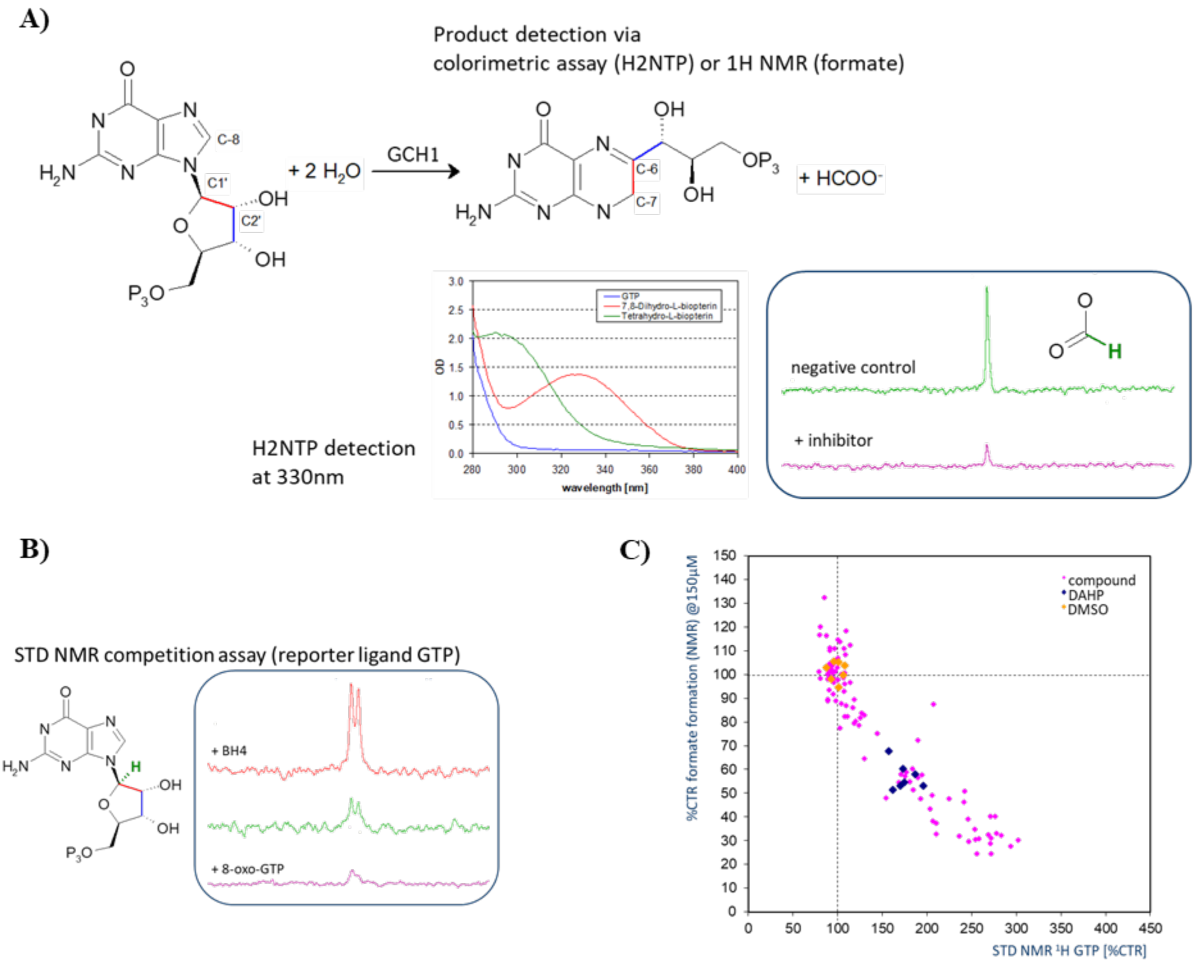

Supplemental Figure 6: Detection of product formation and STD NMR assay. A) Enzyme activity assay for GCH1 based on colorimetric detection of H2NTP or 1H-NMR detection of formate. No H2NTP or formate is produced on presence of inhibitor (e.g. BH4). **B)** STD NMR is capable to detect GTP binding (green). Addition of another active site binder (8-oxo GTP) leads to loss of the signal (purple). STD NMR enhancement of substrate GTP in the presence of allosteric regulator BH4 (red). This screening assay allows for the identification of allosteric binders. **C)** Correlation of activity in functional NMR assay and STD NMR enhancement of active site reporter ligand GTP.
